# Fracture Toughness of Polypropylene-Based Particulate Composites

**DOI:** 10.3390/ma2042046

**Published:** 2009-11-30

**Authors:** David Arencón, José Ignacio Velasco

**Affiliations:** Centre Català del Plàstic, Universitat Politècnica de Catalunya, Edifici Vapor Universitari, Colom 114, 08222, Terrassa, Spain; E-Mail: david.arencon@upc.edu (D.A.)

**Keywords:** polypropylene, composites, fracture toughness

## Abstract

The fracture behaviour of polymers is strongly affected by the addition of rigid particles. Several features of the particles have a decisive influence on the values of the fracture toughness: shape and size, chemical nature, surface nature, concentration by volume, and orientation. Among those of thermoplastic matrix, polypropylene (PP) composites are the most industrially employed for many different application fields. Here, a review on the fracture behaviour of PP-based particulate composites is carried out, considering the basic topics and experimental techniques of Fracture Mechanics, the mechanisms of deformation and fracture, and values of fracture toughness for different PP composites prepared with different particle scale size, either micrometric or nanometric.

## 1. Introduction

Particulate filled polymers are used in very large quantities in all kinds of applications and despite the overwhelming interest in advanced composite materials, considerable research and development is done on particulate filled polymers even today. Fillers increase stiffness and heat deflection temperatures, decrease shrinkage and improve the appearance of composites [1,2,3]. Productivity can also be increased in most processing technologies due to their decreased specific heat and increased heat conductivity [1,2]. Fillers are often introduced into the polymer to create new functional properties not possessed by the polymer matrix at all like flame retardancy [4] or conductivity [5].

The properties of particulate-filled composites are determined by component properties, composition, structure and interaction between phases [6]. Considerable effort has been put on the study of the dependence of mechanical properties on these variables [7,8,9]**.** Within the mechanical properties, fracture toughness is of special relevance on the design of components. Characterisation of the toughness of particulate-filled composites can provide more of a challenge, as a result of the heterogeneity of the compound itself. Traditionally toughness has been characterized by the Izod or Charpy impact energy [7]. It has been long recognized that the impact energy is a very complicated strain rate function of the plastic and fracture work, with generally the plastic work dominating. The Izod and Charpy tests have lost favour in mechanical engineering because they cannot be directly applied in design, but they still have use for comparing the toughness of a particular polymer-particle system. In their desire to characterize toughness of ductile polymer composites more exactly, many researchers have turned to Fracture Mechanics theories [10,11,12].

In this review we focus our attention on the work performed on the determination of fracture toughness of filled systems with polypropylene as thermoplastic matrix. Polypropylene is among all polymers, one of the most researched due to its attractive price/performance ratio, good processing and great recyclability among other factors, so it has found a wide range of applications in household goods, packaging, and automobile industry [13,14,15].

However, owing to its poor impact resistance, especially under extreme conditions such as low temperatures or high strain rates, the usefulness of PP as an engineering thermoplastic is still limited. In order to overcome this inconvenient, attempts have been carried out through the addition of a rubbery phase, but this normally implies a decrease in stiffness, which may not fulfil the product requirements [16,17,18]. A better balance between stiffness and toughness, in some cases, may be provided by particulate fillers and thus fracture toughness of particulate-filled polypropylene has been a challenge that has provoked considerable interest, which is reviewed in the present paper.

## 2. Deformation and Fracture Mechanisms in Polypropylene-Based Materials

The basic deformation mechanism of unmodified polypropylene is shear yielding [19,20], although crazing has been observed in some cases. Shear yielding leads to a permanent change in the dimensions or shape and implies translational motions of the polypropylene chains, reaching great deformations because of the molecular entanglements which act as resistant points. Shear yielding can be observed in a localized o diffused way, depending on the magnitude of the zone affected by this process.

**Figure 1:**
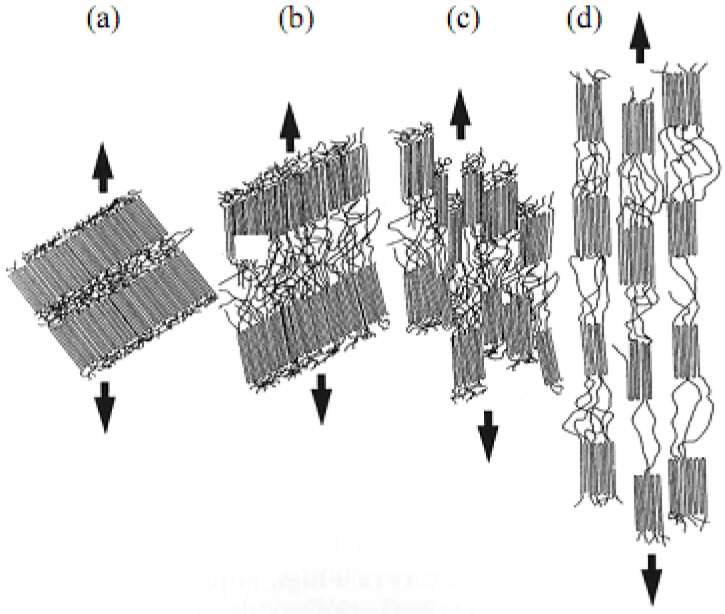
Schematic representation of the deformation process in PP: (a) no deformation, (b) chain motion inside lamellae, (c) lamellae fragmentation and (d) tension alignment (adapted from [20]).

In semicrystalline polymers such as PP, shear yielding is a process localized in the vicinity of the crystalline areas, as described in Figure 1**.** The existence of amorphous zone allows the crystal to show slight distortions (e.g., rotation, shear and intralamellar slipping) with reversible characteristics. The increase of deformation provokes that the localized deformation of the crystals is more marked, leading to a destruction of lamellar aggregates and a irreversible rearranging of the polymer chains. Finally the amorphous zones and the crystals are oriented in the tensile direction leading to a fibrous structure. The localized shear yielding can also be manifested as a consequence of inhomogeneities and instabilities of geometrical origin, superficial and/or internal defects, which take place in the deformation process, promoting the concentration of plastic deformation.

**Figure 2:**
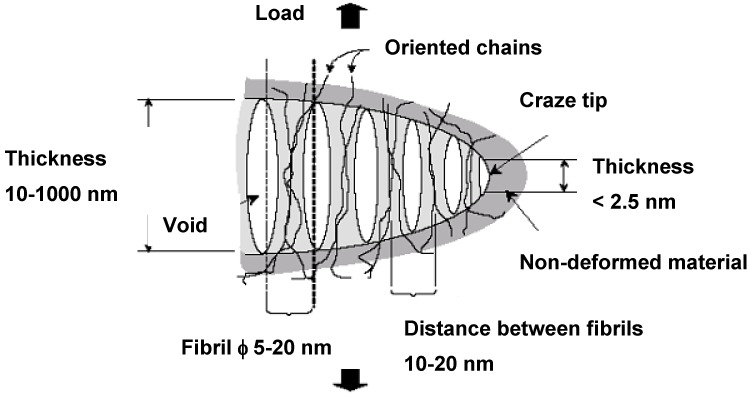
Representation of the ideal structure of a craze.

Crazes can be observed in PP [21,22]. Crazing consists of the generation of a system of interpenetrated microvoids (Figure 2), which are developed on the perpendicular plane to the main tensile direction. These microvoids are stabilized by microfibres of material that does not coalesce. The microfibres in the craze act as bridges in a microcrack, allowing the load to be transmitted, stabilizing the craze and giving rise to an enhancement of resistance. The rupture of the fibrils commonly leads to the microvoid coalescence and thus leading to cracks and ulterior fracture in a brittle manner.

It should be taken into account that the semicrystalline nature of PP implies that the fracture behaviour of polypropylene is strongly dependant on the crystalline structure and superstructure (crystalline form, lamellae dimension, crystallite size, crystallinity, spherulite size) [23,24,25] along with the molecular characteristics (molecular mass and its distribution) and processing-induced morphology.

Introduction of fillers into a polypropylene matrix results in a heterogeneous system. Under the effect of an external load these heterogeneities induce stress concentrations, the magnitude of which depends on the geometry of the particles (mean diameter and average size distribution), on the relative properties of the components and on interfacial adhesion [26]. Heterogeneous stress distribution and local stress maximums developing in the composite influence its deformation and fracture behaviour as well as its overall performance. Also, any influence on the crystalline structure produced by the filler addition will have an influence on the fracture behaviour and deformation mechanisms of the composite.

The incorporation of rigid particles into the polypropylene matrix leads to differences in the overall process of crack propagation and fracture. The process starts with the plastic deformation of the matrix ahead of the initial crack. The micromechanisms leading to the plastic deformation are the debonding of particles (creating holes) and the further plastic flow of the matrix zones remaining between the cavities. These zones are locally stretched until rupture by tearing. In several works, debonding is believed to be the initial damage mechanism. Debonding is especially important in PP composites; because of the low polarity and consequently low surface free energy of this polymer, interfacial adhesion is usually weak, and separation of the matrix –filler interface takes place. Several criteria are proposed for the initiation of the debonding mechanism [27,28,29].

**Figure 3:**
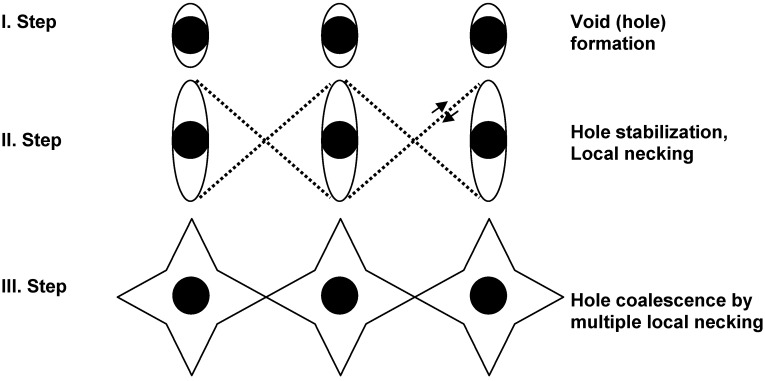
Stages of crack formation around rigid particles in a ductile matrix (adapted from [30]).

The failure sequence of a particulate filled composite is showed in Figure 3. Assuming a poor bonding between filler and matrix, the filler detaches easily from the matrix by creating voids (step I). With further plastic deformation, these voids grow in the stress direction, forming dimple-like holes around the particles (step II). In the next stage of loading, the rest of the matrix deforms under shear conditions until the previous holes coalesce and final fracture occurs. The debonding in step I obviously depends on the filler shape (stress concentration effect) and on the filler-matrix adhesion. The strain levels in steps II and III are considerably reduced by increasing the filler volume fraction, *V_f_*, and thus with decreasing the interparticle distance. This leads finally to a ductile-brittle transition. Of course, the stretching ability of the matrix in steps II and III is controlled by both interparticle distance and the deformability of the matrix.

Coming back to Figure 3, it can be stated that the toughness characterization of filled systems depend on matrix voiding, initiated by the rigid inclusions, and matrix shear deformation around the particles, as well as on the onset and course of these processes (consecutive and/or competitive). The latter are clearly time-dependent. The presence of a plastic zone affects not only the fracture initiation but also the propagation values (e.g. crack bifurcation, deviation [31]). Crack bifurcation in a filled system with coarser particles may generate a zig-zag path and thus enhances the tearing modulus. Crack bowing and pinning may be a further energy absorption mechanism. The crack pinning plays a much more important role in filled or toughened thermosets of very low inherent toughness [32].

When a cracked specimen of a filled PP is loaded in tension, the stress concentrates at the crack tip. Decohesion processes (interfacial debonding, dewetting) take place in this field and a plastic zone appears. In this way, the local stress is reduced (stress-relieving or crack tip blunting). Thus the voided plastic zone becomes more prone to crack penetration and growth. The net effect of the “benefitial” crack blunting and the “detrimental” weakening caused by voided (sometimes termed “cavitation”) depends on which mechanism prevails at the testing conditions. The testing conditions may also influence the blunting process by adiabatic heating at the crack tip [33].

However, it is possible to modify the interfacial adhesion between PP and rigid inclusions, using several approaches: (a) soft rubbery interphase (‘encapsulation’ of the particles in the compounding process [34,35]); polarity decrease of the filler (*i.e.* surface treatment of the fillers by tensile surfactants, chemical coupling agents, etc.[33,36,37,38]); polarity increase of the PP matrix (*i.e.* use of grafted PP, incorporation of ethylene/polar monomer copolymers and the like [39,40]); (d) arbitrary combination of the above methods [41,42]. In case of good interfacial adhesion, the extension of debonding is reduced and as a consequence, the plastic deformation of polypropylene is less extensive.

## 3. Topics of Fracture Mechanics

The use of a stress analysis in modern design procedures ensures that in normal service very few engineering components fail because they are overloaded. However, weakening of the component by such mechanisms as corrosion or fatigue-cracking may produce a catastrophic fracture and therefore in some instances, the fracture properties of the component are the most important consideration. The study of how materials fracture is known as Fracture Mechanics and the resistance of a material to fracture is colloquially known as “toughness” [10,11,12].

**Figure 4:**
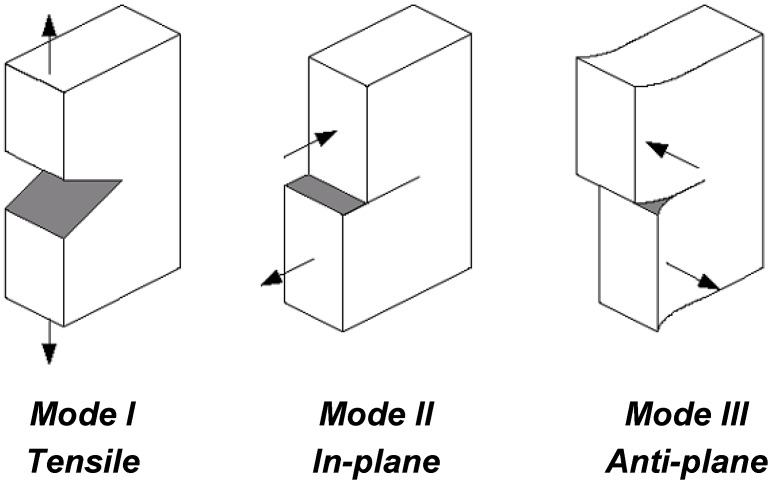
Fracture modes.

No structure is entirely free of defects and even on a microscopic scale these defects act as stress-raisers which initiate the growth of cracks. The theory of Fracture Mechanics therefore assumes the pre-existence of cracks and develops criteria for the catastrophic growth of these cracks. In a stressed body, a crack can propagate in a combination of the three opening modes shown in Figure 4. Mode I represents opening in a purely tensile field while modes II and III are in-plane and anti-plane shear modes respectively. The most commonly found failures are due to cracks propagating predominantly in mode I, and for this reason materials are generally characterized by their resistance to fracture in that mode. The theories examined in following sections will therefore consider mode I only but many of the conclusions will also apply to modes II and III.

Fracture can also be phenomenologically classified according to macroscopic deformation before fracture into three categories [43]: brittle, semi-brittle (or semi-ductile) and ductile fracture. Fracture without any macroscopic plastic deformation or fracture in the elastic state prior to yielding is called brittle fracture. Semi-brittle fracture accompanies local plastic deformation around stress concentrators such as notches or inclusions. Ductile fracture occurs after uniform plastic deformation.

**Figure 5:**
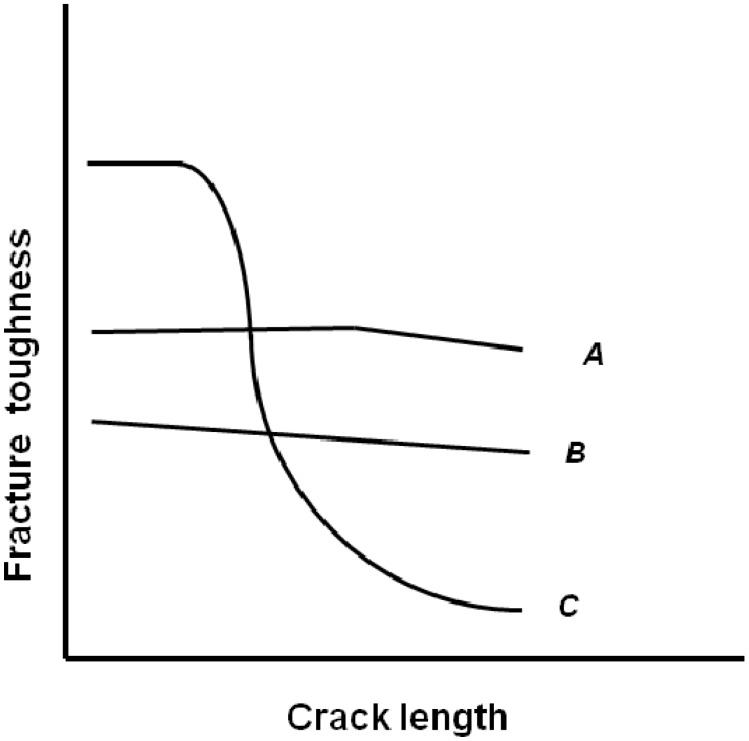
Material classification according to the influence of the crack length on the fracture toughness (adapted from [43]).

Another description of these characteristics is the dependence of fracture resistance on the size of a notch or crack, as shown if Figure 5. The brittle material C exhibits rather high fracture strength without a notch or crack, but the reduction of strength is considerable with an increasing notch depth. The material B exhibits a constant strength no matter what the notch depth. This is ductile fracture independent of the presence of a notch or crack. The strength of the material A remains nearly constant up to a certain critical notch depth and then decreases with an increasing notch depth. This fracture mode, which is in between brittle and ductile fracture, is called semi-brittle or semi-ductile.

### 3.1. Linear Elastic Fracture Mechanics (LEFM)

Griffith [44] considered that fracture produces a new surface area, and that for fracture to occur, the increase in energy required to create the new surface must be balanced by a decrease in elastically stored energy in the sample. To explain the large discrepancy between the measured strength of materials and that based on theoretical considerations, he postulated that the elastically stored energy is not distributed uniformly throughout the specimen or sample, but is concentrated in the neighbourhood of small cracks. Fracture occurs due to these cracks which originate from pre-existing flaws.

**Figure 6:**
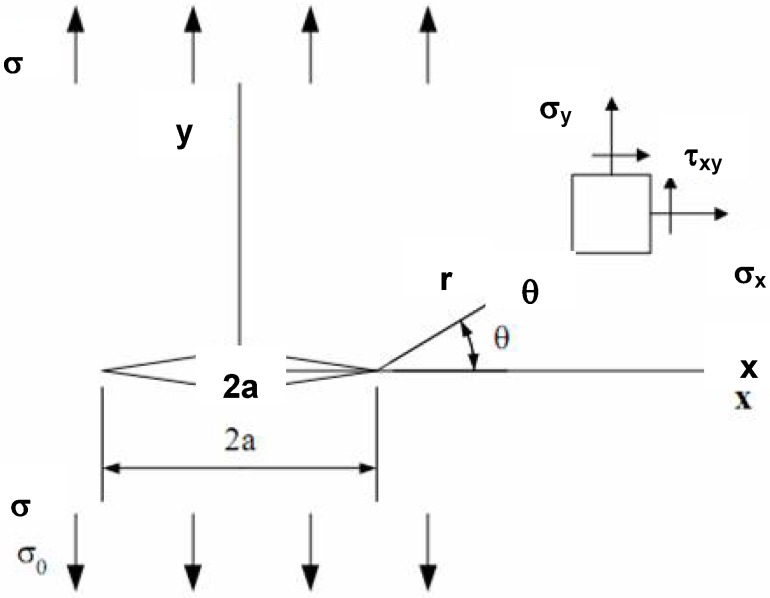
Scheme of a crack of length *a* in an infinite body. *σ* and *τ* denote tensile and shear stresses respectively.

The growth of any crack is usually associated with an amount of work, *dW*, being done on the system by external forces and a change, *dU*, in the elastically stored energy, *U*. The difference between these quantities, *dW-dU*, is the energy available for the formation of a new surface. A crack (Figure 6) of length, *a*, grows when:

(1) dW/da−dU/da≥γ dA/da

where *γ*is the surface free energy per unit area and, *dA*, the associated increment of surface. If there is no change in the overall extension when the crack propagates, *dW*=0 and:

(2) $$-(dU/da)\geq \sigma_{F}dA/da$$

Equation 2 allows the fracture stress, *σ_F_*, of a material to be defined in terms of the crack length by the relationship:

(3) $$\sigma_{F}={\left(2\gamma \;{\text{E}}^{*}/\pi \;a\right)}^{1/2}$$

where E^*^ is equal to the Young’s modulus, *E*, for a thin sheet under plane stress conditions and to *E/(1-υ ^2^)*, where *υ*, is the Poisson ration, for a thick sheet in plain strain conditions. For an infinite sheet with a central crack of length, *a*, subjected to a uniform stress *σ*, Irwin showed [10] that:

(4) $$K=\sigma {\left(\pi \;a\right)}^{1/2}$$

Irwin postulated that when *σ* reaches the fracture stress, *σ_F_*, *K* takes a critical value:

(5) $$K_{c}=\sigma_{F}{\left(\pi \;a\right)}^{1/2}$$

where the fracture toughness of the material can then be defined by the value of *K_c_*, called the critical stress intensity factor which defines the stress field at fracture zone. Equation 5 can be written as:

(6) $$\sigma_{F}={\left(K_{c}^{2}/\pi \;a\right)}^{1/2}$$

being identical in form to Equation 3, which is Griffith’s formulation. The strain energy release rate, *G*, is the energy available for a unit increase in crack length (Equation 7). Fracture occurs when *G* reaches a critical value *G_c_*, and *G_c_* is equal to 2*γ*in Griffith’s formulation.

(7) G=dW/dA−dU/dA

**Figure 7:**
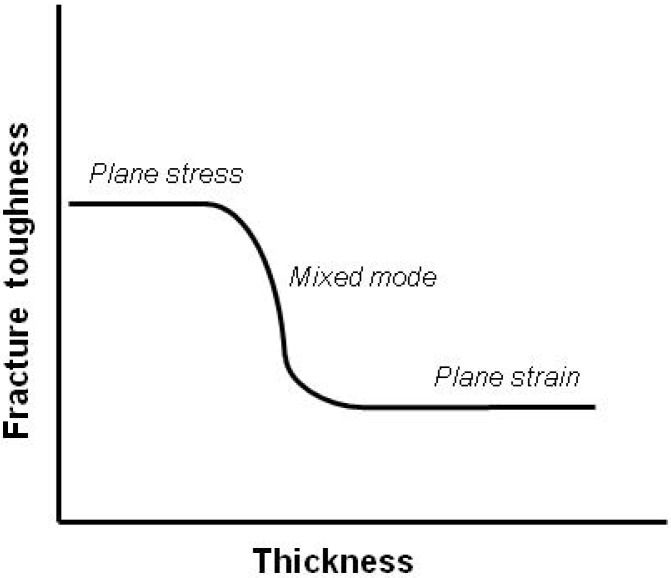
Influence of specimen thickness on the fracture toughness values and related stress states (adapted from [19]).

Generally, in plane stress conditions, the plastic zone crack tip is produced by shear deformation through the thickness of the specimen. Such deformation is enhanced if the thickness of the specimen is reduced. However, if the specimen thickness is increased then the additional constraint on through-thickness yielding produces a triaxial stress distribution so that approximate plane strain deformation occurs with shear in the *xy* plane. There is usually a transition from plane stress to plane strain conditions as the thickness is increased (Figure 7). As *K_c_* values are generally quoted for plane strain, it is important that this condition prevails during fracture toughness testing. In mode I of fracture (tensile) a well-established criterion for plane stress conditions is that the thickness, *B*, should obey the following:

(8) $$B\;\geq \;2.5\;\frac{{\left(K_{IC}\right)}^{2}}{\sigma_{y}^{2}}$$

Where *K_Ic_* is the critical intensity stress factor for mode I and *σ_y_* is the tensile yield stress. It should be noted that, even on the thickest specimens, a region of plane stress yielding is always present on the side surfaces because no triaxial stress can exist there. The greater plasticity associated with the plane stress deformation produces the characteristic ‘shear lips’ often seen on the edges of fracture surfaces. In some instances the plane stress regions on the surfaces may be comparable in size with nominally plane strain regions and a mixed-mode failure is observed. However, many materials show a definite transition from plane stress to plane strain.

Since polymers are viscoelastic materials, strict LEFM theory cannot be applied and the total work of fracture must include the various forms of plastic deformation, which appear prior to and during failure [7,19,45]. In particular, three points should be considered:

(a) Because of the relatively low yield stress values of many plastics, plastic deformation at crack tip is far more likely to occur.
(b) While a small degree of dissipative energy can be accommodated in the overall work of fracture, it is obvious that as this assumes greater significance, there is much greater possibility that a fracture mechanics approach will lose its general validity.
(c) Plastic properties such as fracture toughness and yield stress are dependent on many variables related to fracture testing, and for a given material, the test conditions necessary to ensure validity are therefore quite restricted.

The bottom line is that the major problem in applying LEFM theory to polymers is to assess the extent to which the plastic deformation zone at the crack tip influences the resulting fracture behaviour.

### 3.2. Elastic-Plastic Fracture Mechanics (EPFM)

The problems associated to the LEFM analysis on polymers have led to the development of several elastic-plastic fracture analyses, being the most employed approaches the *J*-integral and the Essential Work of Fracture (EWF).

#### 3.2.1. The *J*-integral concept

**Figure 8:**
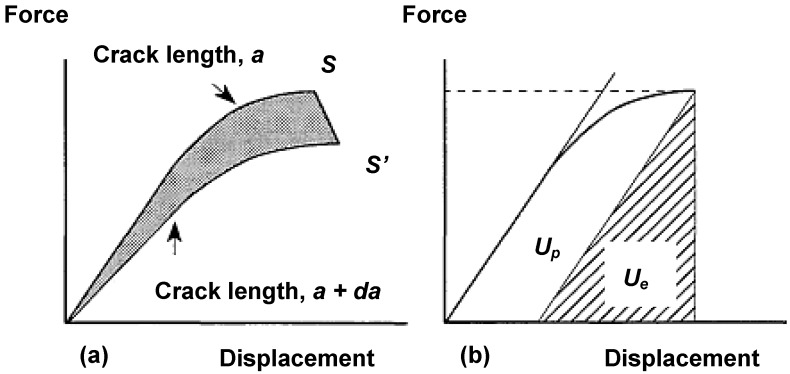
Elastic-plastic behaviour. **(**a) Decrease of the potential energy due to crack growth; (b) Separation of the elastic and plastic contributions.

*J*-integral was originally defined by Rice [46] as a contour integral independent on the path, which express the energy per unit area necessary to create new fracture surfaces in a loaded body containing a crack. From load-displacement curves of two bodies with initial crack lengths *a* and (*a+da*), as indicated in Figure 8, if the crack propagation takes place in point *S* and *S’* for the first and second body respectively, the area between the two curves (shadowed zone) is the energy necessary to produce a crack surface.

This can be expressed as:

(9) $$J=-\frac{1}{B}\left(\frac{dU}{da}\right)$$

The resulting fracture criterion is *J* ≥ *J_c_*, being *J_c_* a critical value independent on both the crack length and the sample geometry. Sumpter and Turner [47] expressed Equation 9 as:

(10) $$J=J_{e}+J_{p}=\frac{\eta_{e}U_{e}}{B(W-a)}+\frac{\eta_{p}U_{p}}{B(W-a)}$$

where *J_e_* and *J_p_* are the elastic and plastic contributions of the whole *J* value. *U_e_* and *U_p_* are also the elastic and plastic components of the total energy *U*. Moreover, *η_e_* and *η_p_* are the elastic and plastic work factors respectively. *J* is defined in the same terms as *G*, that is, as energy per unit area of crack propagation. The critical value *J_c_* is compatible with LEFM and equivalent with *G_c_* for stable fracture, tough in *J* is assumed that the load-displacement curves are independents on the path, which is not strictly correct. In tough materials, such as polypropylene, the crack tip gets blunted previously to the crack propagation process. As traditionally used in the field of metals, a blunting line can be defined to estimate the crack blunting before the crack propagation:

(11) $$J=2\;\sigma_{y}\Delta \;a$$

#### 3.2.2. Essential Work of Fracture (EWF)

**Figure 9:**
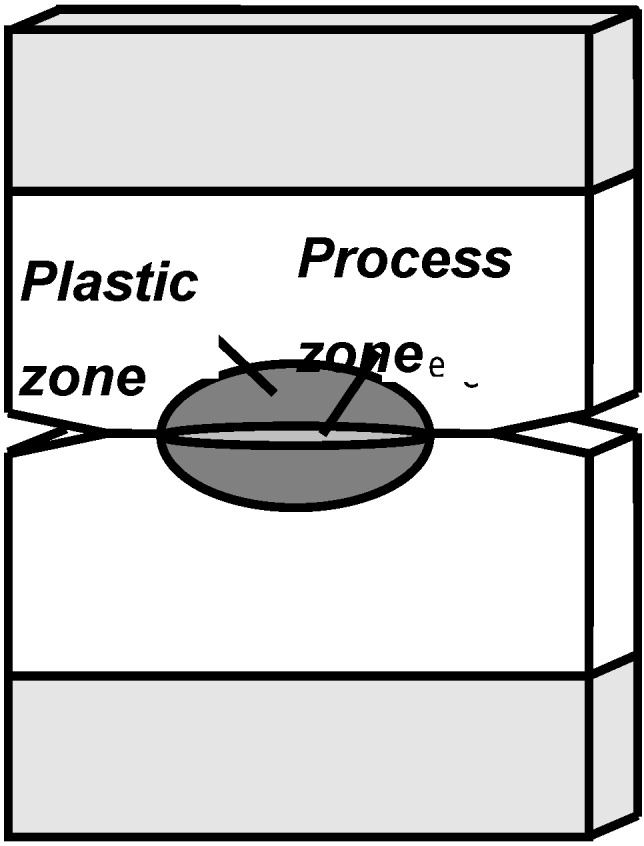
Process and plastic zones involved in a notched specimen under tension.

The theory of EWF was initially developed by Broberg [48], and is based on the consideration that, when a notched specimen is loaded in tension, the total work (*W_f_*) involved in fracture is dissipated in two distinct zones, called the inner (or process) and the outer (or plastic) zones (Figure 9).

Broberg postulated that *W_f_* could be divided into two terms: the essential and the non-essential work of fracture (*W_e_* and *W_p_* respectively). The first item is related to the instability of the crack tip, where the real fracture process occurs, and it is proportional to the ligament section (*lt*), while the second one is associated with the plastic work, and considered proportional to the plastic zone volume (*β l^2^t*):

(12) $$W_{f}=W_{e}+W_{p}=w_{e}lt+w_{p}\beta \;l^{2}t$$

where *w_e_* is the specific essential work of fracture (per surface unit), *w_p_* is the non-essential specific work of fracture (per volume unit), *l* is the ligament length, *t* is the specimen thickness and *β* a shape factor of the plastic zone. Dividing this equation by the ligament section, we have:

(13) $$w_{f}=w_{e}+\beta w_{p}l$$

This concept is very useful in the characterization of fracture behaviour of films and sheets, geometries which do not fulfil the size requirements of both LEFM and *J*-integral.

## 4. Experimental Procedures to Determine Fracture Parameters

### 4.1. Linear Elastic Fracture Mechanics Testing

**Figure 10:**
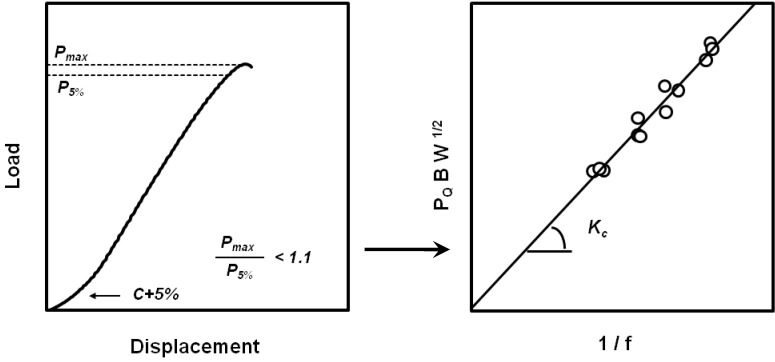
Determination of the critical stress intensity ratio, *K_c_*, value from experimental load-displacement curves.

**Figure 11:**
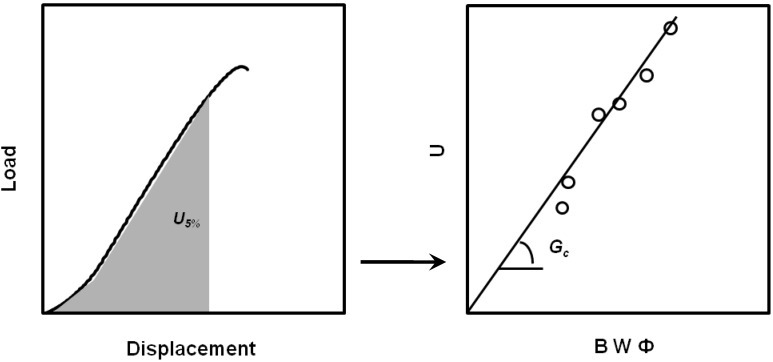
Determination of the critical strain energy release rate, *G_c_*, value from experimental load-displacement curves.

Several LEFM standard testing protocols for determining *K_c_* and *G_c_* for plastics are found in the literature [49,50,51,52,53], taking into account several specimen geometries and strain rates. Once a specimen has been prepared according to the protocol specifications, the specimen is loaded to failure while recording the load-displacement curve (Figure 10). For tests in which the load of fracture, defined by *P_Q_*, meets the standard’s requirements, a preliminary toughness value, *K_Q_*, is calculated from the appropriate stress intensity solution. It is accepted that *P_Q_* is *P_max_* when the compliance (*C*) plus 5% *C* intercepts the load-displacement curve at a load value lower than *P_max_*; otherwise, *P_Q_* corresponds to *P_5%_*. Thus, having obtained *K_Q_*, the specimen size requirements must be validated to assure plain strain conditions, and only then can *K_Q_* be called *K_c_*. The integration of the curve load-displacement will lead to the value of *G_c_* (Figure 11). Dimensionless factors ( *f* and *Ф*) are obtained from the theoretical development and depend on the specimen geometry and fracture mode.

### 4.2. J-R Resistance Curve Determination

**Figure 12:**
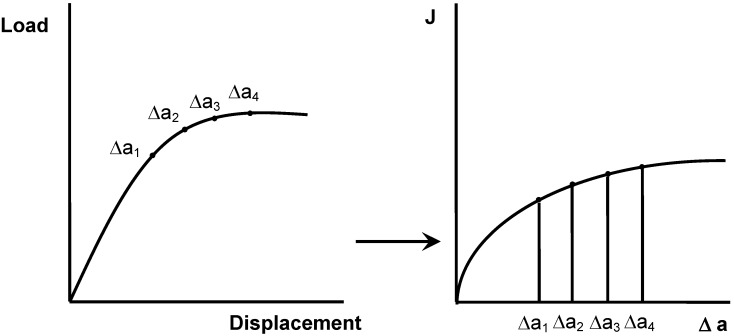
Construction of *J-R* curves from load-displacements curves obtained at different crack displacements.

*J*-integral determination is usually performed by using a multiple specimen methodology that allows to obtain the called *R*-curve, according to the procedures recommended by [54,55]. To construct the *R*-curve (*J - Δa*), a set of identical specimens is loaded monotonically to different deflections, all less than that to give total failure, to obtain different levels of stable crack extension, and then fully unloaded (Figure 12).

**Figure 13:**
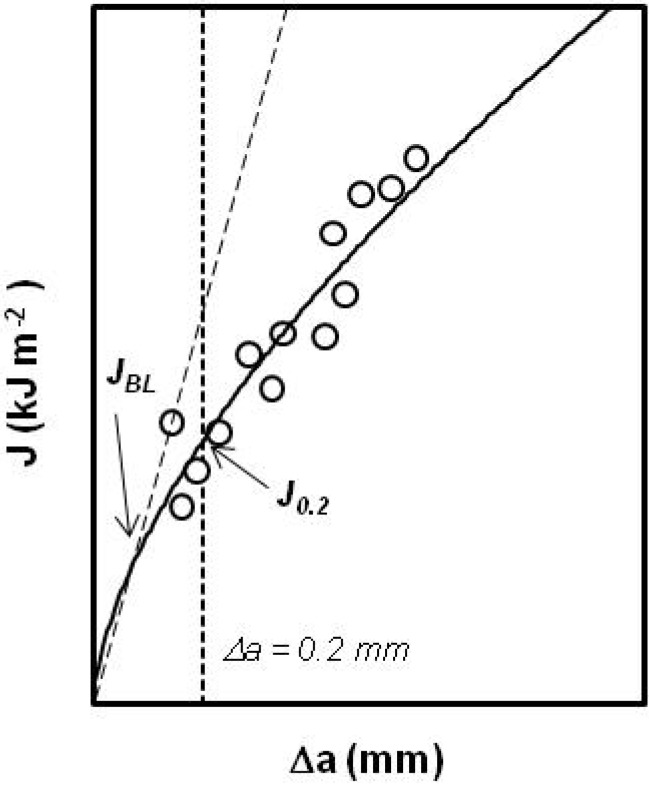
Determination *J_0.2_* and *J_BL_* from the experimental *J-R* curves.

Crack growth initiation occurs at a critical value of the *J*-integral, *J_c_*, and in tough materials like. In PP-based materials an initial pseudo-extension of crack occurs, which is due to crack tip blunting. To consider this effect a crack blunting line is traditionally used. The initiation fracture toughness, *J_Ic_* (Figure 13) is defined [54] as the lower value of a *J_0.2_* parameter or a *J_BL_* value (specified as the intersection of the blunting line with the *J-R* curve, that is, *J_Ic_* = min {*J_0.2_, J_BL_*}.

An alternative way to obtain *J* critical values is the normalization method, which allows the construction of the resistance curve from the record of a single fracture test. This method is based on the assumption that load can be separated into two independent and multiplicative functions, namely the geometry function and the deformation function, which depend only on the crack length and the plastic displacement, respectively. The work of Sharobeam and Landes [56] resulted in the definition of a criterion, which allows the load separation validity to be experimentally checked. Once load separation has been checked and the deformation function is determined, the value of the crack length increment can be determined at any instant of the test and the *J- Δa* curve can be plotted.

### 4.3. Essential Work of Fracture Testing

**Figure 14:**
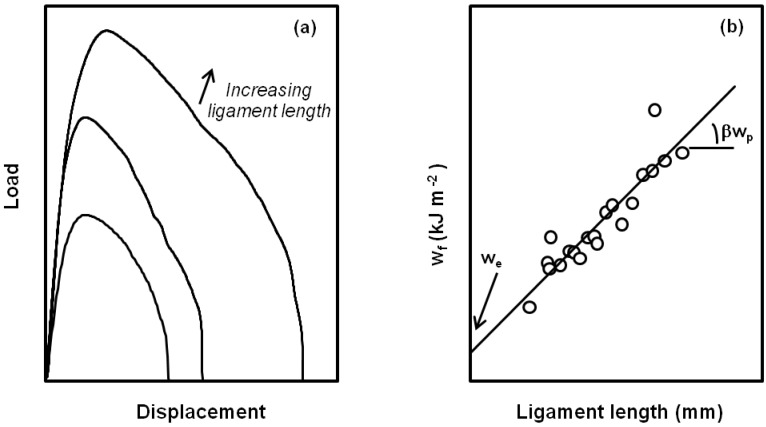
Determination of EWF parameters (*w_e_* and *w_p_*) from load-displacement curves with increasing ligament lengths.

The EWF theory proposed by Broberg was experimentally developed by Cotterell and Mai [57] for plastic materials. A testing protocol proposed by Clutton [58] is nowadays employed in the study of fracture toughness of films and thin sheets of polymers. A certain number of pre-cracked specimens (the most employed geometry is DDENT, (deeply double edge notched tension)) with different ligament lengths are loaded in tensile mode. The analysis of the energy under the load-displacement curves obtained (Figure 14a), allows to obtain a linear fitting, and getting the values of the specific essential work of fracture and non-essential (or plastic) work of fracture (Figure 14b).

### 4.4. Impact Tests: Charpy, Izod, Falling Weight

The viscoelastic nature of polypropylene implies that its deformation and fracture behaviour is dependent on the strain rate [10,59]. High speed tests, through the application of impact techniques, whether pendular (Charpy, Izod) or axial (falling weight), are strongly relevant to characterize the fracture of this material, as the fracture conditions can be assumed to those of a part in service. The impact resistance is evaluated in terms of energy per unit area, and the energy that better defines the impact resistance is that absorbed by the sample during its deformation and rupture.

When using analogical equipments, the total energy registered in the impact test is the sum of several contributions: the stored elastic energy of the equipment, the absorbed energy by the equipment as a result of vibrations after the initial contact between the striker and the sample, the spent energy by the deformation type Brinell on the sample load points; the energy absorbed by the sample during its fracture and deformation (flexion, initiation and crack propagation), and the energy consumed to accelerate the sample parts once fractured.

These disadvantages of analogical tests can be partially overcome in instrumented tests, [60,61] where the striker (and is some cases the support basis) is equipped with load sensors that allows to separate the different energy contributions and to obtain the value of the energy absorbed by the sample due to the processes of deformation and fracture.

## 5. Fracture Toughness Characterization of PP Microcomposites

### 5.1. PP Composites with Spherical Particles

#### 5.1.1. Glass beads

Solid glass microspheres are a special type of filler that induces improved processability and service performance in injection-moulded polypropylene-matrix composites. Higher thermal conductivity, dimensional stability and small and well-distributed internal stress are three qualities of the glass microsphere-filled PP composites.

Sjöngren *et al*. [62] focused on the failure mechanisms of these composites with glass beads surface-treated with an aminosilane. It was found that during deformation until total fracture, debonding cracks are opened and transformed to cylindrical entities parallel to direction, leading during this process to a significant reduction of the matrix stiffness. Larger diameter beads led to the formation of a large defect and subsequent brittle crack growth, whereas with smaller beads, the matrix fails in a ductile tearing process. Asp *et al*.[63] studied the same failure initiation process by finite element analysis. Residual thermal stresses were demonstrated to have a large effect on global failure initiation stress. Cavitation always occurred at a higher global stress than debonding. However, higher residual stresses than estimated could lead to yield initiated failure although experimental data did not support this. Instead data as well as the results from modelling study were in support of debonding as the initial failure mechanism. These findings [62] correlated with the obtained values of Izod impact strength and drop weight impact energy (Table 1), being lower in composites with larger particles and higher filler volume content.

The same dependence on the glass bead content was found by Tsui *et al*. [64], as an increase in the filler concentration led to a decrease in the values of the fracture toughness of the composite. Liang *et al*. [65] studied the drop weight-impact behaviour of polypropylene with different filler concentrations and sizes. At higher filler concentration, the values of the maximum impact load and the crack initiation energy for the PP filled with smaller glass beads were higher than those of the unfilled PP and the systems filled with larger glass beads. The influence of the filler content and size on the impact fracture of these composites was significant. Comparatively, the drop-weight dart impact resistance of the systems filled with smaller glass beads was somewhat improved.

**Table 1:** Notched Izod strength and drop weight impact energy of PP-glass bead composites (data extracted from [62]).

Material*	Filler (vol. %)	Izod strength (kJ m ^-2^)	Drop weight energy (J)
neat PP	0	no break	5.89
A5	4.8	12.5	5.98
B5	4.8	10.9	5.81
A20	19.3	7.2	5.65
B20	21.0	6.1	1.43

*A composites: glass beads with medium diameter of 3.5-7.0 μm. *B composites: glass beads with medium diameter of 27-36 μm.

**Figure 15:**
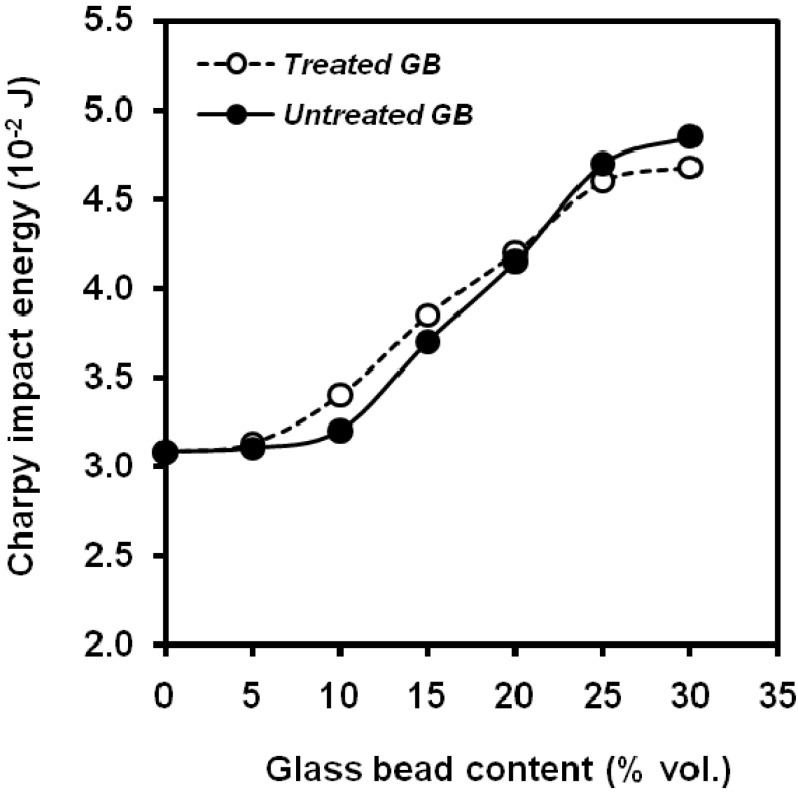
Dependence of the notched Izod fracture energy on the glass bead content. Particle medium size was 35 μm. Treated glass beads were covered by CP-03 silane. (adapted from [66]).

**Figure 16:**
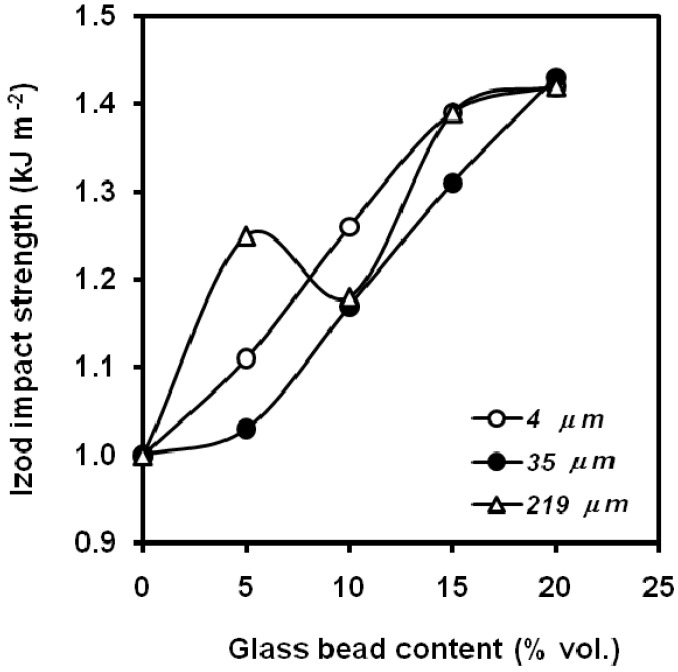
Dependence of the notched Izod fracture energy on the glass bead content for composites with several particle sizes (adapted from [67]).

However, Liang *et al*. [66] observed an increase in the notched Izod impact energy (Figure 15) from a glass bead content of 10 vol.% with no influence of the surface treatment and found also an increasing trend with several particle sizes (Figure 16) [67], explained on the basis that when glass bead filled PP composites receive an impact force, the beads will induce crazes in the matrix around the surface because of stress concentration and will increase surface area to absorb the impact fracture energy. At the same time, the beads play a role in hindering the propagation of the crack. Dubnikova *et al*. [68] explained the ductile-brittle transition on the basis of the impeding of adhesion failure microprocesses led to the ductile-brittle transition at low degrees of filling. The impact strength of composites with weak adhesion non-monotonically varied with an increase in the filler content, and the toughness rose at a filler content of 10-15 vol.% It was shown that the enhancement of cracking resistance in the case of facilitated debonding of rigid particles is due to pore initiation and energy loss for yielding at the crack initiation step. The conservation of inclusion-matrix bonding upon loading decreases the toughness of the filled polymer. A model for evaluating the fracture toughness of filled polymers, which takes in account the influence of debonding stress on the energy loss process upon impact loading was proposed. Also an increasing trend [69] in Izod impact properties was found when silane-treated hollow glass beads (11, 35 and 70 μm medium size) were used, noticing a maximum in 15wt.% of filler. The highest value was obtained with the smallest particles.

**Figure 17:**
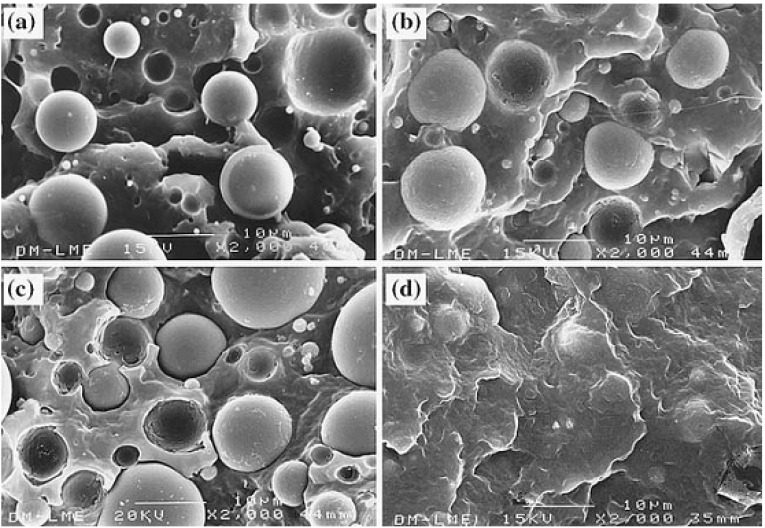
Degrees of interfacial adhesion achieved by the modification of the glass bead surface and the polarity of PP matrix. (a) Low adhesion, (b) and (c) medium adhesion, (d) high adhesion (adapted from [40]).

In contrast to that showed by [69], the modification of the interfacial adhesion through the surface treatment of glass beads with *N*-(2-(vinylbenzylamino)-ethyl)-3-aminopropyl trimethoxysilane led to a fall in the Izod impact strength [70]. The combination of glass bead surface treatment and modification of PP polarity gave a wide range of degrees of interfacial adhesion (Figure 17), as shown by Arencón *et al*. [40], who studied the fracture toughness of composites with high filler content (26 vol.%). The fracture toughness characterization techniques were selected according to this extent of plastic deformation and therefore, *J*-integral and EWF (Table 2), and LEFM (Table 3), concepts were employed. At low strain rate composites with medium and high interfacial adhesion displayed quasi-brittle fracture, although only the latter fulfilled LEFM requirements. Composites with both grafted maleic anhydride polypropylene (MAPP) and glass beads treated with aminosilane showed the highest value of *K_Ic_* and *G_Ic_*. Brittle fracture was observed in all the studied composites at moderate impact speed (0.5 m s^-1^). The fracture of composites with low interfacial adhesion level was ductile at low strain rate (1 mm min^-1^) and it could be studied through *J*-integral analysis and EWF. The addition of PET resulted in lower fracture toughness, due to the poor compatibility between both polymers.

**Table 2:** J-R curve and EWF analysis of PP-glass bead composites with 26 vol.% of particles (data extracted from [40]).

Matrix	Surface treatment	J-R curve analysis		EWF analysis	
		J_Ic_ (kJ m ^-2^)	J_0.2_ (kJ m ^-2^)	w_e_ (kJ m ^-2^)	βw_p_ (MJ m ^-3^)
PP	untreated	3.5	5.3	12.2	3.6
	A-189	3.3	5.2	11.0	3.5
	Z-6020	2.3	3.0	10.1	2.7
PP/PET (95/5)	untreated	0.6	1.5	8.3	2.3
	A-189	0.8	1.5	9.9	2.5
	Z-6020	1.0	2.0	10.8	3.3

**Table 3:** LEFM analysis of PP-glass bead composites with 26 vol.% of particles (data extracted from [40]).

Matrix	Surface treatment	Low strain rate		High strain rate	
		K_Ic_ (MPa m^1/2^)	G_Ic_ (kJ m ^-2^)	K_Ic_ (MPa m^1/2^)	G_Ic_ (kJ m ^-2^)
PP	untreated	—	—	2.5	1.9
	A-189	—	—	2.5	2.1
	Z-6020	—	—	2.4	1.3
	Z-6032	—	—	2.4	1.1
PP/MAPP (97/3)	untreated	—	—	2.6	1.5
	A-189	—	—	2.2	1.0
	Z-6020	2.2	1.4	2.8	1.6
	Z-6032	2.4	1.8	2.9	2.1
PP/PET (95/5)	untreated	—	—	2.4	1.3
	A-189	—	—	2.3	1.3
	Z-6020	—	—	2.5	1.6
	Z-6032	—	—	2.3	1.0
PP/MAPP/PET (92/3/5)	untreated	1.7	0.9	2.3	1.0
	A-189	1.7	0.9	2.1	0.9
	Z-6020	2.2	1.2	2.8	1.8
	Z-6032	2.3	1.5	3.1	2.2

#### 5.1.2. Calcium carbonate

Among the many possible fillers for plastics, calcium carbonate (CaCO_3_) fulfils most of the requirements expected for its use in polypropylene: it is a plentiful mineral filler, cheap, with a large variety of purity and size particle, generally with globular shape, its surface may be properly coated and besides of the low price, the mechanical properties and shrinkage post-moulding of the polymer are improved. Therefore, it’s not surprisingly that many researches on this kind of PP-based composites have been performed.

**Figure 18:**
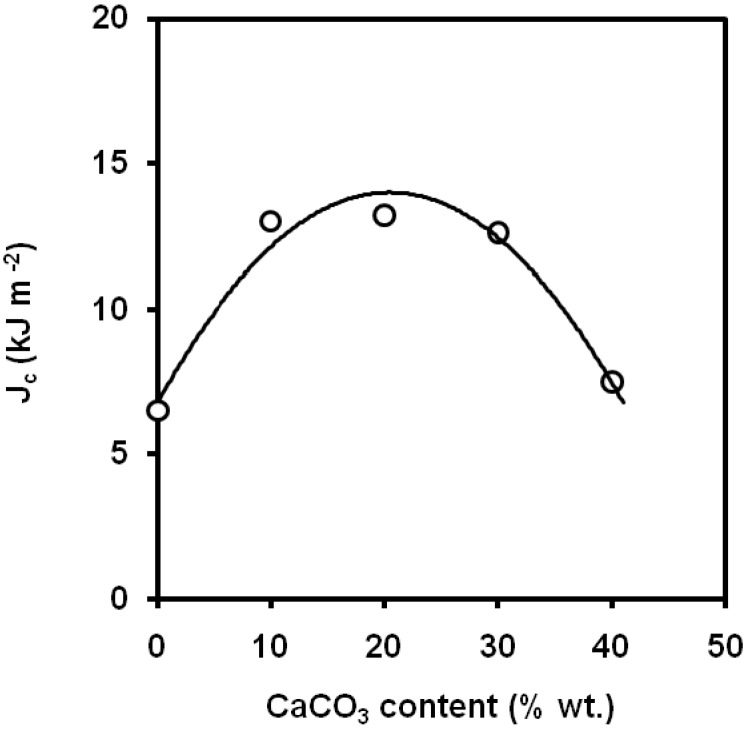
Influence of CaCO_3_ content on the values of *J_c_* (adapted from [71]).

**Figure 19:**
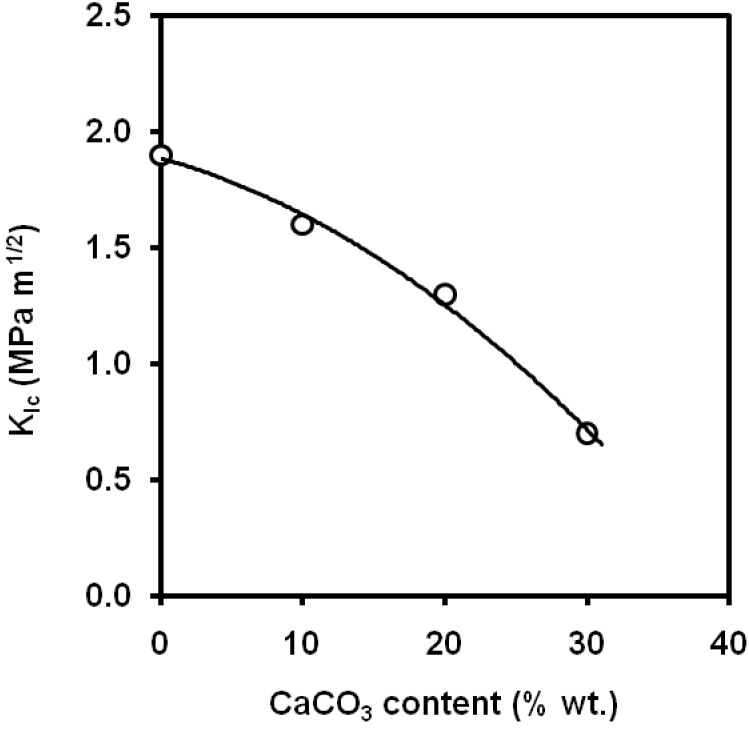
Influence of CaCO_3_ content on the values of *K_Ic_* (adapted from [74]).

Li *et al*. [71] applied the *J*-integral concept to characterize the fracture toughness of PP/CaCO_3_ composites at low strain rate. The addition of calcium carbonate led to a change in the fracture mode from brittle fracture for virgin PP to ductile fracture for the filled composites, which was attributed to the changes of stress fields in the PP matrix around the filler particles. The particle/matrix debonding occurs at low stress level and promotes the yielding of the PP matrix microligaments. As a result, the fracture resistance was increased up to 20wt.% of calcium carbonate (Figure 18). Pukánszky *et al*. [72] also report in impact tests a maximum in the values of *G_c_* at a calcium carbonate volume fraction of 0.2. An increasing trend within the range 0-30 wt.% of calcium carbonate was found [73] in the values of notched Izod fracture energy. By other hand, Zerbajad *et al*. [74] showed that the higher content of CaCO_3_ the lower values of *K_Ic_* (Figure 19) obtained by 4 point bending tests. Leong *et al*. did not found significant differences in the values of notched Izod impact strength within the range 0-0.16 of volume fractions for composites containing untreated CaCO_3_ particles with average medium size of 3 μm [75].

**Figure 20:**
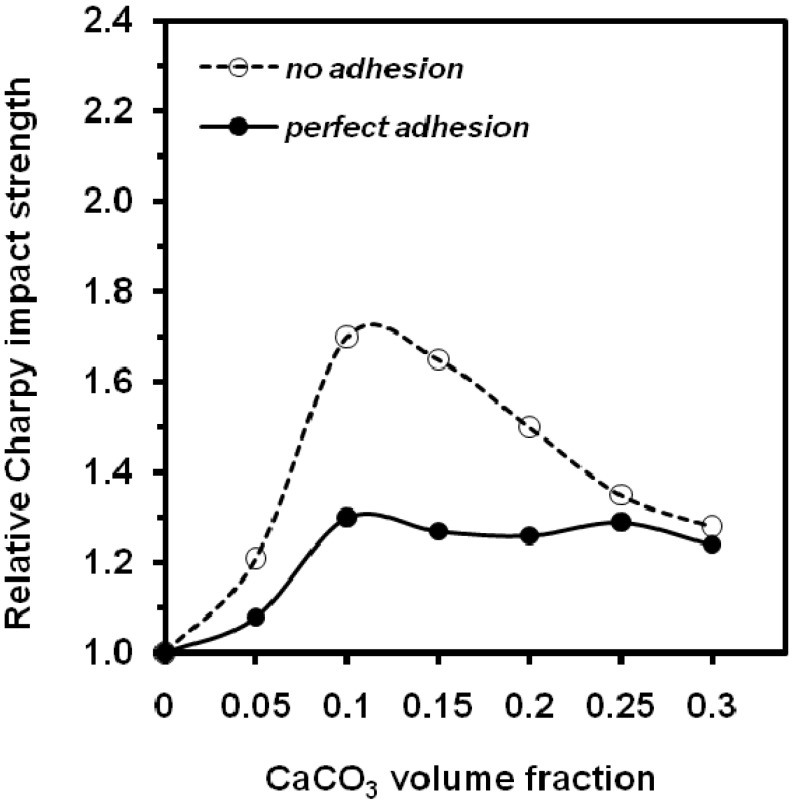
Influence of CaCO_3_ on the relative (composite/matrix) notched Charpy impact strength (adapted from [76]).

**Figure 21:**
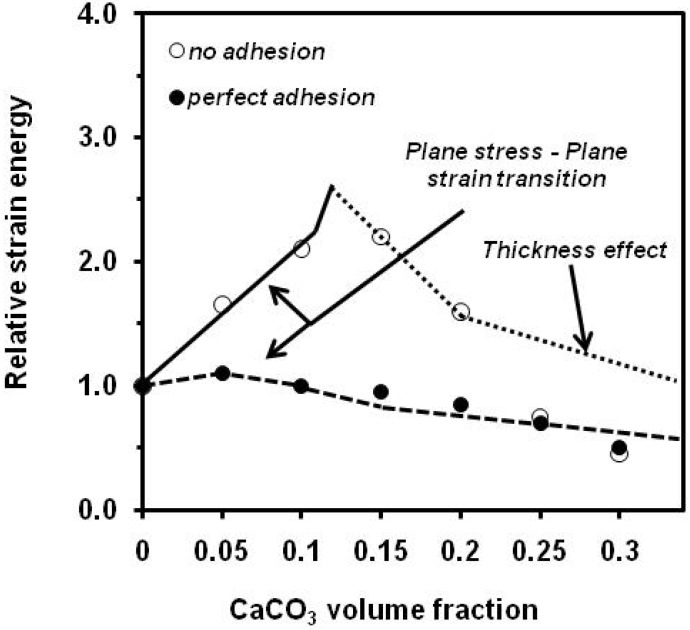
Influence of CaCO_3_ on the relative (composite/matrix) critical strain release energy (adapted from [76]).

Jancar *et al*. [76] studied the concentration dependence of Charpy impact strength (Figure 20) and *G_c_* (Figure 21) and in terms of competition between the effects of increasing stiffness, decreasing effective matrix cross section, and the transition from plane strain to a plane stress mode of failure. In the case of no adhesion between components, the size of the crack tip plastic zone increases with increasing filler volume fraction because of the reduction of the material yield strength. In the region 0 < *V_f_* < 0.12, there is a mixed mode of failure, and the measured value of *G_c_* for crack initiation increases steadily as the sample cross section approaches a fully plane stress state. The reduction in yield strength also results in the increase in *G_c_* for crack propagation as reflected by an increase in Charpy impact strength. Above *V_f_* = 0.12, the specimen cross section is in a fully plane stress state, and further increase in filler volume fraction (decrease in matrix effective cross section) has the net effect of reducing both *G_c_* and impact strength. In the case of ''perfect'' adhesion, the yield strength increases only slightly with filler volume fraction. In the region 0 < *V_f_* < 0.05 there is also a mixed mode of failure, but the increase in *G_c_* is much less than that for the no-adhesion case since the size of the plastic zone in front of the crack is much smaller. Above *V_f_* = 0.05, the combined effects of increasing stiffness, reduction of the size of the plastic zone, and decreasing matrix cross section dominate the behaviour, causing a steady reduction in both *G_c_* and impact strength. Good agreement was found between experimental data and calculations based on Fracture Mechanics principles.

The study of the modification of the interfacial adhesion degree between PP and CaCO_3_ through the variation of the matrix polarity was also analysed by Gong *et al*. [77, 78] using the EWF methodology. The specific work of fracture of PP/CaCO_3_ composites was appreciably lower than that of pure PP, while the displacement to failure and the total plastic energy dissipation decreased markedly with increasing CaCO_3_ content (Table 4). For the PP/CaCO_3_ composites modified with MAPP, we increased remarkably at first and then decreased with increasing amount of MAPP, while the displacement to failure decreased considerably.

**Table 4:** EWF parameters obtained from PP-CaCO_3_ composites with several ratios of MAPP. (adapted from [77]).

PP/CaCO_3_ /MAPP (mass ratio)	w_e_ (kJ m ^-2^)	βw_p_ (MJ m ^-3^)
100/0	21.82	11.57
90/10	18.30	10.11
80/20	19.56	8.97
70/30	19.33	7.01
80/20/3	23.72	7.48
80/20/5	19.54	7.67
80/20/7	16.56	6.99

A beneficial effect of a pinellic acid-surface treatment was found [79], on the Izod impact strength based on the improved interfacial adhesion between matrix and filler and the promotion of the crystallization of polypropylene in form of β-spherulites. tougher than α-spherulites [24]. Zuiderduin *et al*. in notched Izod [73] and Kucera *et al*. in notched Charpy [80] impact tests, observed, higher values of fracture energy, when the surface of calcium carbonate particles were treated with stearic acid. A liquid titanate also proved its effectiveness in increasing the values of notched Izod impact strength within the range 0-30 vol.% of calcium carbonate [81].

**Figure 22:**
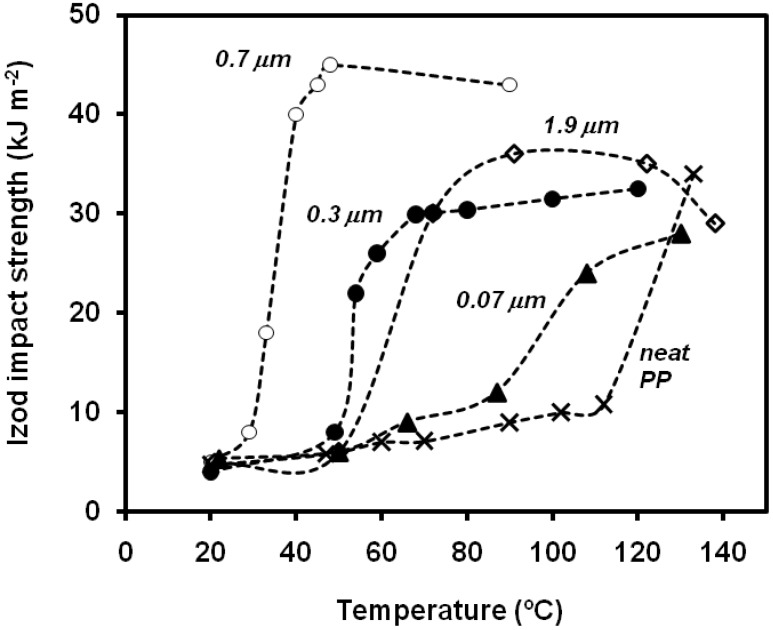
Influence of particle size and temperature on the values of notched Izod impact strength of PP-CaCO_3_ composites (adapted from [73]).

As expected, the filler size plays also a decisive role. Thio *et al*. [82] found that filler size had a key effect on improvement of impact strength of PP. Only 0.7-μm diameter fillers improved Izod impact energy, whereas 0.07 and 3.5 μm diameter fillers had either an adverse or no effect on the impact toughness. Zuiderduin *et al*. [73] also observed that, among four different sizes of calcium carbonate particles (0.07, 0.3, 0.7 and 1.9 μm), the 0.7-mm diameter CaCO_3_ fillers treated with stearic acid at testing temperatures close to 40 ºC (Figure 22) gave the best combination of properties. Yang *et al*. [83] found an optimum for 0.07 medium size when comparing the notched Izod impact strength of several PP/CaCO_3_ composites (0.07, 1.8, 4 and 25 μm), and also reported differences in the trends of the impact energy *vs.* the filler content when the PP matrix was varied.

**Figure 23:**
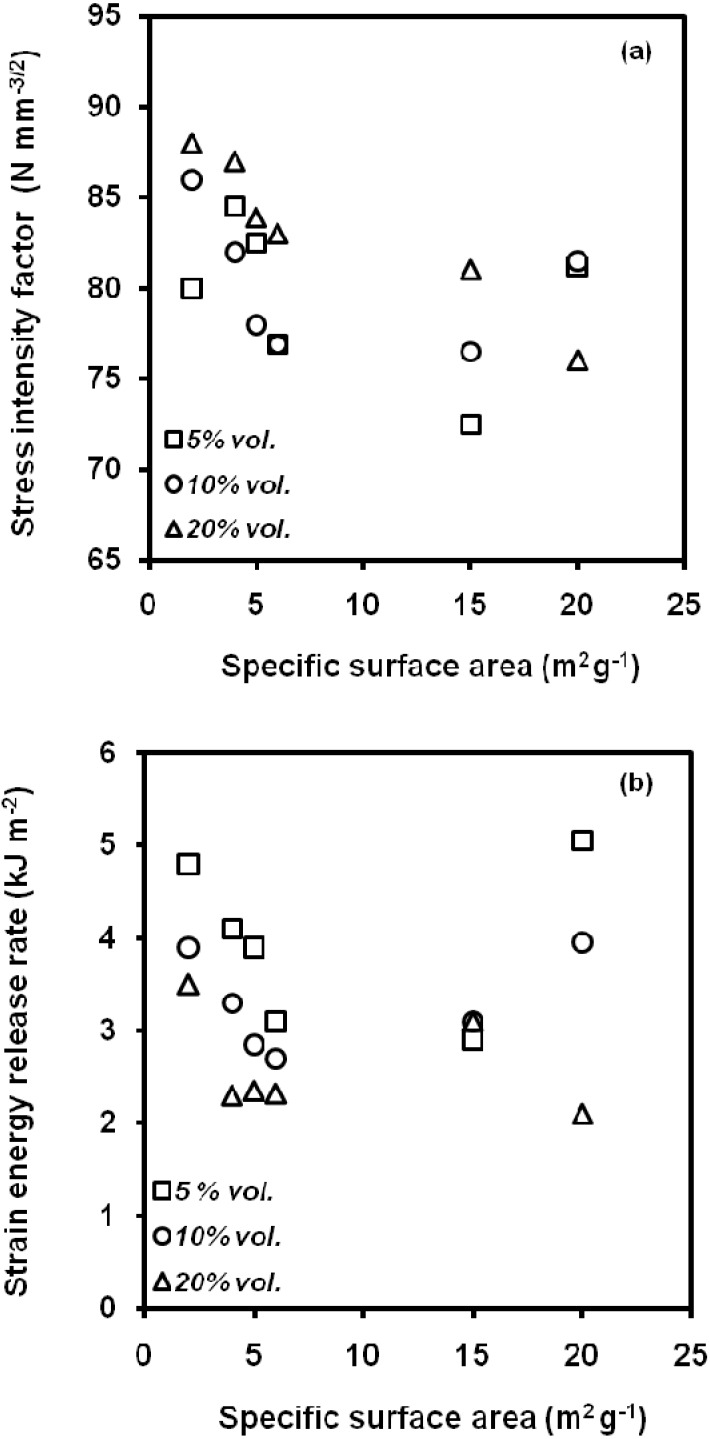
Influence of the specific surface area of CaCO_3_ particles on the values of (a) the stress intensity factor, *K_c_*, and (b) the critical strain release rate, *G_c_*, of PP-CaCO_3_ composites (adapted from [84]).

The particle size has a direct effect on the dispersion of the particles into the polypropylene matrix. Fekete *et al*. [84] showed that filler aggregation occurred when their particle size was smaller than a critical value, depending on component properties and processing conditions. The impact strength decreased with increasing the number of aggregates, which also influenced on the trend of *K_c_* and *G_c_* values with the specific surface area (Figure 23). In presence of an extensive aggregation of small particles, cracks were initiated inside and propagated through aggregates.

**Figure 24:**
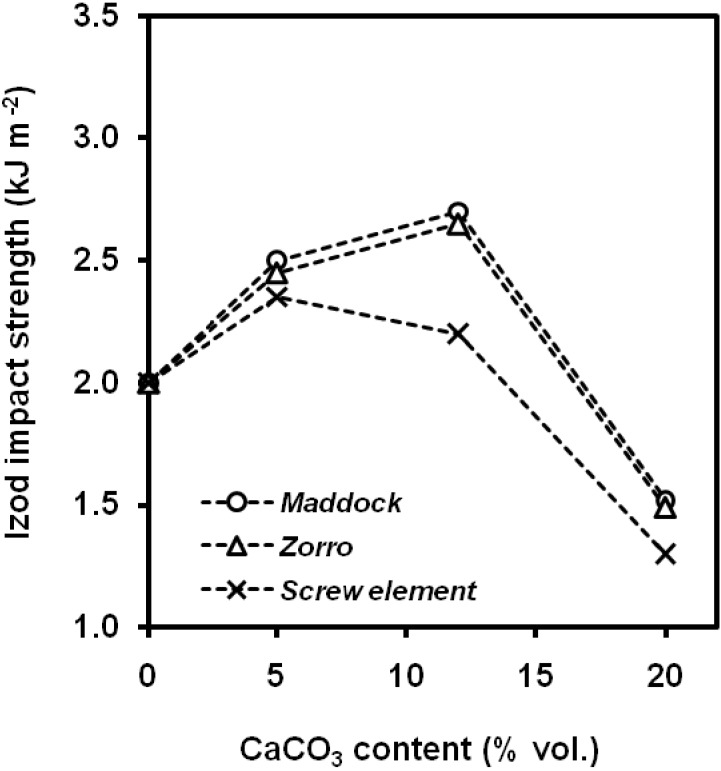
Notched Izod impact strength evolution with silane-treated CaCO_3_ content for several screw configurations (adapted from [81]).

Agglomerates may be reduced with improved dispersion in the processing step. Wang *et al*. [81] studied the influence of the particle dispersion on filled PP-CaCO_3_ composites obtained by single screw extrusion process, with several filler volume fractions (5, 12, 20 vol.%). The particles employed were untreated and surface-treated with a liquid titanate compound. Figure 24 shows the notched Izod impact strength evolution with the filler content, using different screw configurations. A best dispersion was achieved at 12 vol.% in both untreated and surface-treated particles, even better the latter. This led to the highest values of composite toughness. In a later work [85] compared the performance of the coverage of calcium carbonate particles with liquid titanate and stearic acid on the notched Izod impact strength of composites coming from two compounding processes, twin-screw extruder and an internal mixer. For both compounding methods and surface treatments, an optimal CaCO_3_ content (10 vol.%) was found. Stearic acid provided slightly higher values of impact strength than liquid titanate.

Hutar *et al*. [86] studied the toughening of a PP-CaCO_3_ composite using a three-phase finite-element model. Based on the LEFM, the behaviour of a microcrack was estimated for several particle sizes and interphase properties. It was found that one of the basis mechanisms of toughening consisted in shielding the influence of rigid particles by a soft interphase, followed by debonding. This effect was strongly size- and material-dependent and in the case of PP-CaCO_3_, the particle size at which the effect of rigid particles is completely shielded by the soft interphase was smaller than 8-10 μm.

#### 5.1.3. Other fillers

**Figure 25:**
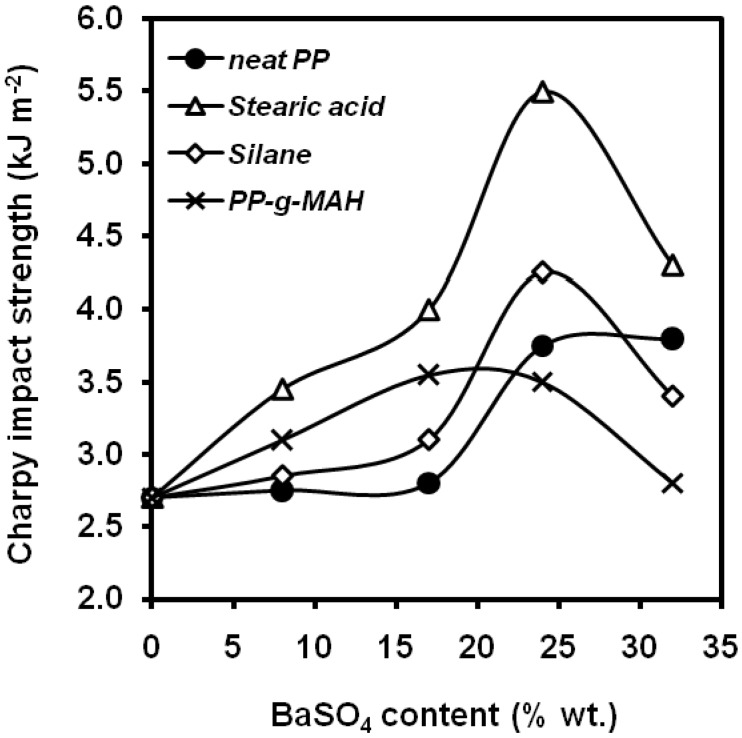
Influence of barium sulphate content on the notched Charpy impact strength of PP composites with differences in matrix-filler interphase (adapted from [87]).

Wang [87] found that PP can be toughened with specially treated BaSO_4_ particles as the interfacial modification contributed to the toughening in two aspects: the first is to provide a proper interfacial adhesion, which ensures the inorganic particles transfer the stress and stabilizes the cracks at the initial stage of deformation and satisfy the stress conditions for plastic deformation of matrix ligaments subsequently via debonding. The second is that the modified interface between PP matrix and BaSO_4_ increased the nucleating ability of the fillers and retards the motion of the PP chains, which led to the formation of PP crystals with less perfection and smaller size in the matrix, promoting plastic deformation of the matrix after the debonding occurs. The stearic acid modified system (Figure 25) showed the highest toughness because of its moderate interfacial adhesion and its crystalline morphology in the matrix. These compositions were also characterized by the EWF methodology [88] reporting that very strong interfacial adhesion was not favourable for toughness, because the debonding-cavitation process may be delayed and the plastic deformation of matrix maybe restrained. The surface treatment of BaSO_4_ with stearic acid provided the highest values of specific work of fracture, *w_e_*.

It was observed in PP/kaolin composites a decrease in the values of the notched Charpy impact strength within the range of 0-40 wt.% of kaolin particles [89]. The surface treatment of kaolin with stearic acid didn’t give any significant improvement, but quaternary ammonium coverage provided remarkable increases of the impact strength, up to 240% in the case of 40 wt.% of kaolin. Also a dramatic increase was observed [90] when kaolin was treated with latex. A beneficial effect of non-reactive surface treatments applied to kaolin on notched Izod impact strength was noticed [91]. Velasco *et al*. [92] reported the effect of particle size in PP composites with aluminum hydroxide, Al(OH)_3_, showing that finer particles provided higher values of *K_Ic_* and *G_Ic_* than coarser ones (Table 5).

**Table 5:** Fracture parameters for PP/AL(OH)_3_ composites (20 vol.%). Average size for OL and ON particles is 1.5 and 60 μm respectively. Data extracted from [92].

Material	*K_Ic_* (MPa m^1/2^)	*G_Ic_* (kJ m^-2^)
PP	2.36	2.40
PPOL	2.43	2.07
PPON	2.22	1.82

Mai *et al*. [93,94] observed in PP/Al(OH)_3_ systems that the improvement of interfacial adhesion through the in-situ functionalization of PP led to an increase of the Izod impact strength of the composite, depending on the content of initiator, dicumyl peroxide and the monomer used to functionalize, acrylic acid. The amount of functionalized PP had also an effect on the impact properties.

### 5.2. PP Composites with Lamellar Particles

#### 5.2.1. Talc

**Figure 26:**
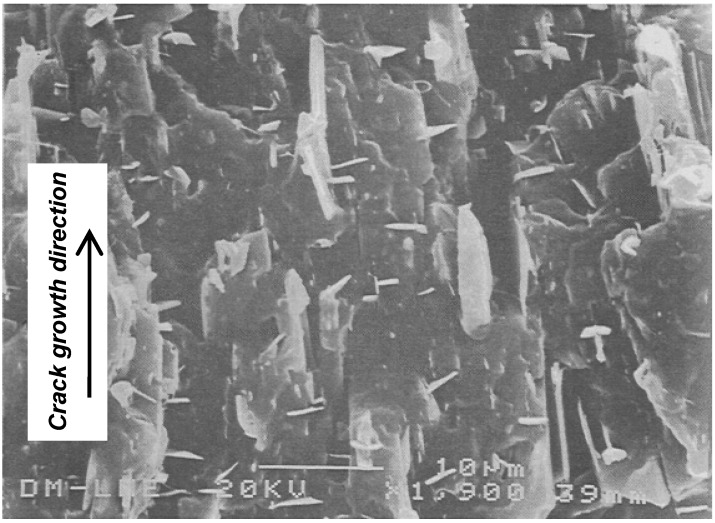
Scanning electron micrograph of a 40 wt.% PP-talc composite, showing the orientation of talc platelets (adapted from [96]).

Talc is along with calcium carbonate, the mineral filler most employed for PP. It is a lamellar phyllosilicate that due to its platy-shaped particle may give a 2D reinforcement in the resulting material. It promotes higher stiffness than calcium carbonate as well higher anisotropy. Velasco *et al*. [95] studied the fracture behaviour of a series of composites of PP filled with untreated and silane-treated talc at low strain and high strain rates. In high strain tests, all the composites failed in a brittle manner; it was found that moderate fractions of talc increased the *K_c_* values of the composite (Table 6) independent of the talc surface treatment; this improvement was attributed to the peculiar orientation of the talc platelets in the injection-moulded specimens (Figure 26); the *G_c_* values achieved its maximum at a talc volume content of 20%. By other hand, at low strain rates the composites developed ductile fracture. Those filled with silane-treated talc presented poor *J*-integral values (Table 6) compared to those of the samples with untreated talc what was related with the reduction of the plastic zone at the crack tip, since the improved coupling between the talc platelets and matrix increased the yield strength of the composites.

**Table 6:** LEFM analysis carried out at impact velocities (0.5 m s^-1^) and J-integral analysis performed on SENB (single-edge notched bend) specimens at 1 mm min^-1^. N and S refers to untreated and silane surfaced-treated talc respectively; the number besides refers to the talc volume fraction in the composites.

Material	LEFM analysis		J-integral analysis	
	K_c_ (MPa m ^1/2^)	G_c_ (kJ m ^-2^)	J_c_ (kJ m ^-2^)	J_0.2_ (kJ m ^-2^)
Neat PP	1.90	2.10	n.d	n.d.
N2	2.02	2.23	3.53	4.39
N10	2.42	2.95	3.67	4.35
N20	2.47	3.11	3.51	4.80
N40	2.63	2.49	2.96	4.06
S2	2.13	2.35	1.85	2.24
S10	2.39	2.81	1.63	1.97
S20	2.69	2.93	1.47	1.85
S40	2.47	1.80	0.85	1.52

**Figure 27:**
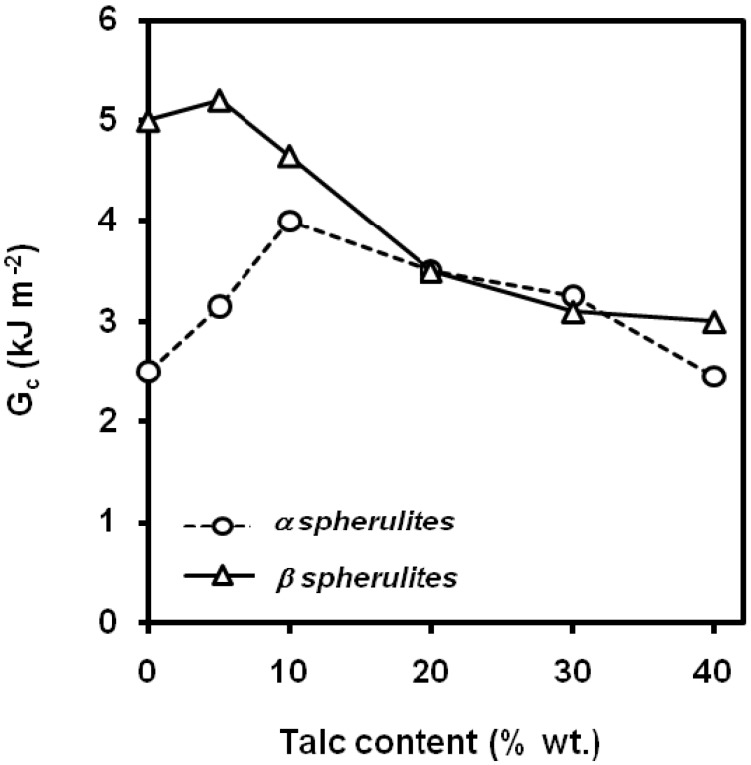
Influence of talc content on the values of *G_c_* of PP-based composites with predominant α or β spherulitic crystalline structure (adapted from [98]).

Some authors have found a maximum in the *G_c_* values with respect to the talc content showed that talc suppresses the formation of β-form PP dramatically (tougher than α-form) [96,97,98]. As a result, the beta-PP composites containing talc content greater or equal to 20 wt.% consisted mainly of the alpha-form of PP. The impact tests revealed that the critical strain energy release rate of the β-PP polymers appears to increase with the addition of 5 wt.% of talc (Figure 27); thereafter it decreases significantly with increasing talc content.

Shelesh-Nezhad *et al*. [99] report a maximum of the notched Izod impact strength at a talc content (untreated) of 20 wt.%, whereas Maiti *et al*. [38] showed a dramatic decrease to 60% of the initial value of the PP matrix at a talc content ca 30 vol.% and Leong [100] reported within the range of volume fractions 0-0.16 a stable Izod impact strength value up to 0.08 volume fraction of talc and then a dramatic decrease. Svehlova *et al*. found [101] that notched Charpy toughness decreased with increasing talc content below matrix level at the highest filler content. In the same work, the effect of talc size was dealt, using four types of talc particles. Notched Charpy toughness decreased according to a linear dependence with mean size of talc particles.

**Figure 28:**
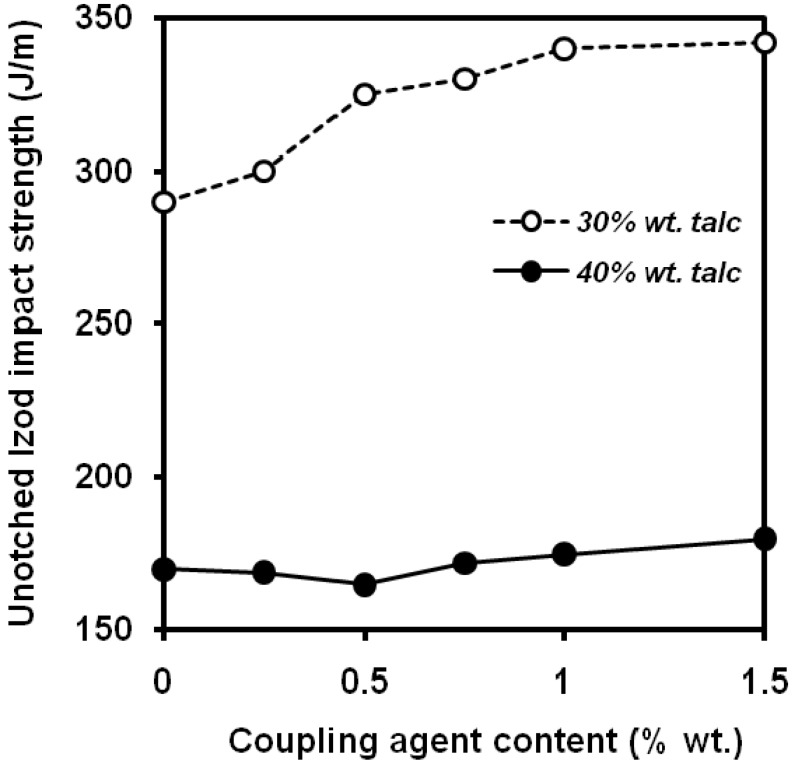
Influence of titanate coupling agent content applied onto the particle surface on the unnotched Izod impact strength values of PP/talc composites (adapted from [102]).

Wah *et al*. [102] studied the effect of the surface coverage of talc with a titanate coupling agent on PP/talc composites. A beneficial effect of titanate coupling agent on toughness was observed (Figure 28), coming from two ways: enhanced interfacial adhesion and plasticizing effect of the low molecular weight substance. Surface treatments based on stearic acid led to higher values of notched Izod impact strength [103] whereas oleic acid had a negative effect.

**Figure 29:**
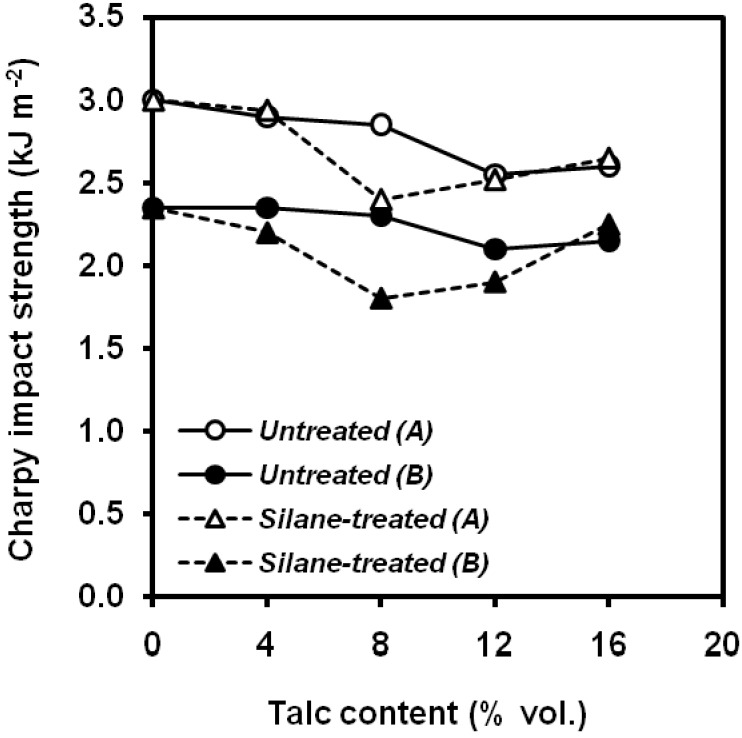
Influence of talc content on the notched Charpy impact strength of (A) non-irradiated and (B) gamma-irradiated PP/talc composites (adapted from [104]).

An interesting study was performed on the influence of gamma radiation of PP/talc composites in order to deep in their capability as final products of sterilized industry. It was found that the gamma-radiation reduced the composite fracture toughness (Figure 29) within all the range of studied compositions. [104]

#### 5.2.2. Magnesium hydroxide

**Figure 30:**
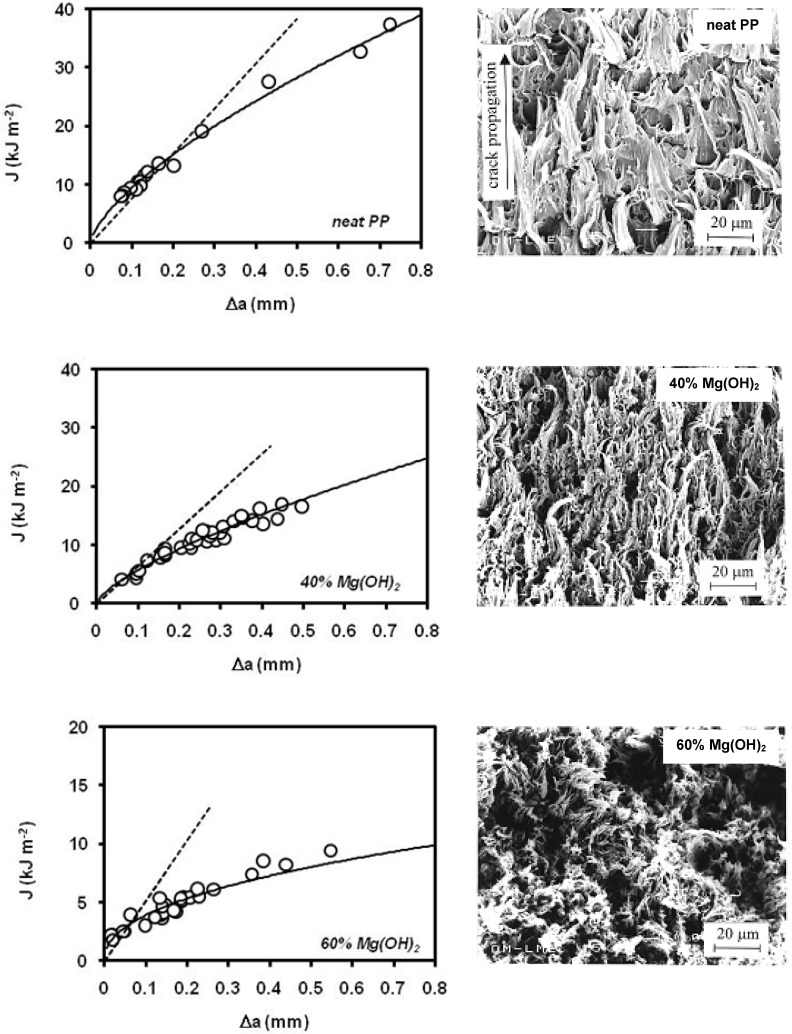
*J-R* curves and fracture surfaces obtained from neat PP and composites with magnesium hydroxide with filler contents of 40% and 60 wt.% (adapted from [105]).

The addition of magnesium hydroxide, Mg(OH)_2_ into a polypropylene matrix aims for a improvement of the flame retardancy of the polymer. Nevertheless, high filler contents are needed to reach these requirements, and thus, fracture toughness of polypropylene, within other mechanical properties, is severely affected. Velasco *et al*. [105] found that the main crack propagation mechanism identified in these materials is ductile tearing, initiated by microvoids nucleation at the interfacial sites. This mechanism is more marked in the filled samples, due to their weak particle/matrix interface. Mg(OH)_2_ particles act as internal defects in the polymer and, as the filler volume fraction is raised, a decrease of the fracture toughness could be observed (Figure 30). Nevertheless, the presence of the filler particles limits to the matrix plastic flow during the material fracture process, which results in higher resistance to crack growth after the fracture onset as the filler volume fraction increases in the composite.

Hornsby *et al*. [106] also found that the filler content affected markedly to the toughness performance of the composite, as the values of impact energy obtained from falling weight impact tests decreased dramatically (up to four times) within the range 0–60 wt.% of magnesium hydroxide. This work also dealt exhaustively with the effect of surface treatment of Mg(OH)_2_ particles with fatty acid derivatives, silane and titanate coupling agents on the mechanical properties of the composites. Silane coupling agents gave some improvements in impact strength, but fatty acid derivatives (Table 7) were found to be by far the most effective surface treatment for improving toughness; this significant improvement was attributed to a modification of the polymer deformation mechanism in the vicinity of the filler particles, resulting in localized voiding, manifested as stress whitening. It was also reported that the level of surface treatment had a significant effect on the values of fracture toughness.

**Table 7:** Impact strength obtained from falling weight tests on PP/Mg(OH)_2_ composites containing 50 wt.% of particles with several surface treatments (adapted from [106]).

*Trade name*	*Chemical name*	*Content ( wt.%)*	*Application temp. (ºC)*	*Impact strength (J 6 mm)*
Z-6070	Methyltrimethyoxysilane	3	18	2.3
Dynasylan	Octyltriethoxysilane	3	18	2.3
Z-6032	(*n*-vinylbenzylaminoethyl)-γ- aminopropyltrimethoxysilane hydrogen chloride	1	18	2.2
Z-6082	Vinyltris (-γ-methoxyethoxy) silane	1	18	2.3
KRTTS	Isopropyltriisostearoyl titanate	3	45	3.2
KR12	Isopropoxy-tris(dioctylphospato) titanate	3	45	2.9
KR38S	Isopropoxy-tris(dioctyl-pyrophospate) titanate	3	45	2.3
KR41B	Tetraisopropoxy-bis(dioctyl-phosphito) titanate	3	45	2.6
TILCOM CA10	Isopropoxy triisostearoyl titanate	3	45	2.8
Hycar HM10	Calcium oxidate soap	10	160	6.2
	Magnesium estearate	10	160	10.3
	Zinc estearate	10	160	10.7
	Stearic acid	10	45	5.9
	Glycerolmonostearate	6	160	5.9
	Azeleic acid	10	45	1.6
	Oleic acid	6	160	1.7

Chen *et al*. [107] reports a good performance of a silane and even better a silicone oil on the values of notched Izod impact energy for PP/Mg(OH)_2_ composites (ratio 1:1).The interfacial adhesion was modified through the increase of the polarity of polypropylene with the addition of functionalized polypropylene and acrylic acid [108] leading to a change in the fracture morphologies of PP/Mg(OH)_2_ composites, as a result of a transformation of the fracture mechanism from debonding of particles into a fracture which develops through the matrix. The addition of MAPP and above all maleic anhydride grafted polyoxyethylene, POE-g-MA, [109] led to an increase in notched Charpy impact energy values.

**Figure 31:**
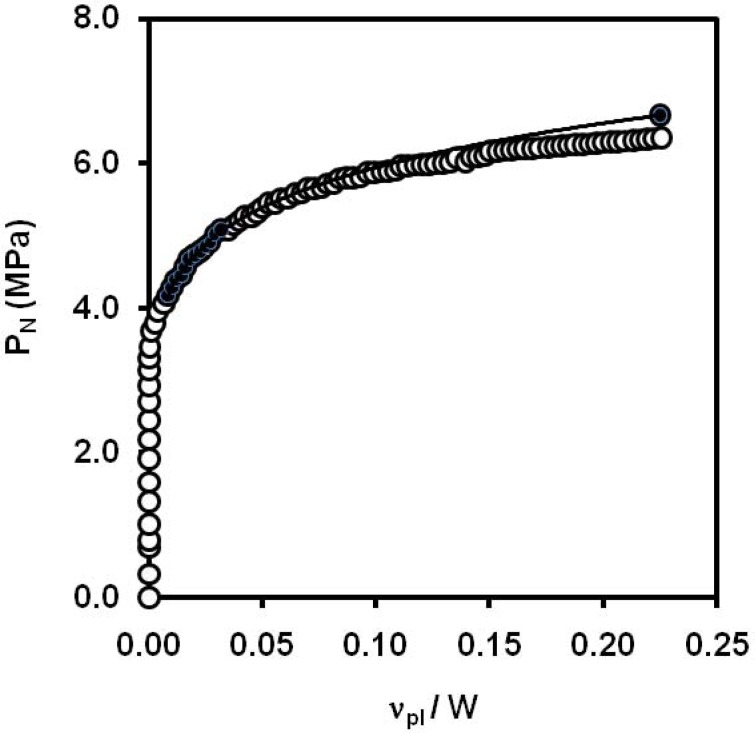
Graphic determination of the limits of the separation blunting region for a PP/Mg(OH)_2_, 40 wt.% composite (adapted from [110]).

Morhain *et al*. [110] applied the concept of load normalization in polypropylene composites with high amounts of magnesium hydroxide (Figure 31). They found that the normalisation method was not applicable to composites containing 60 wt.% of magnesium hydroxide, but for 40 wt.% of particles and neat PP. A good agreement between the multiple-specimen *J-R* curve with the obtained by the single specimen method was obtained.

**Figure 32:**
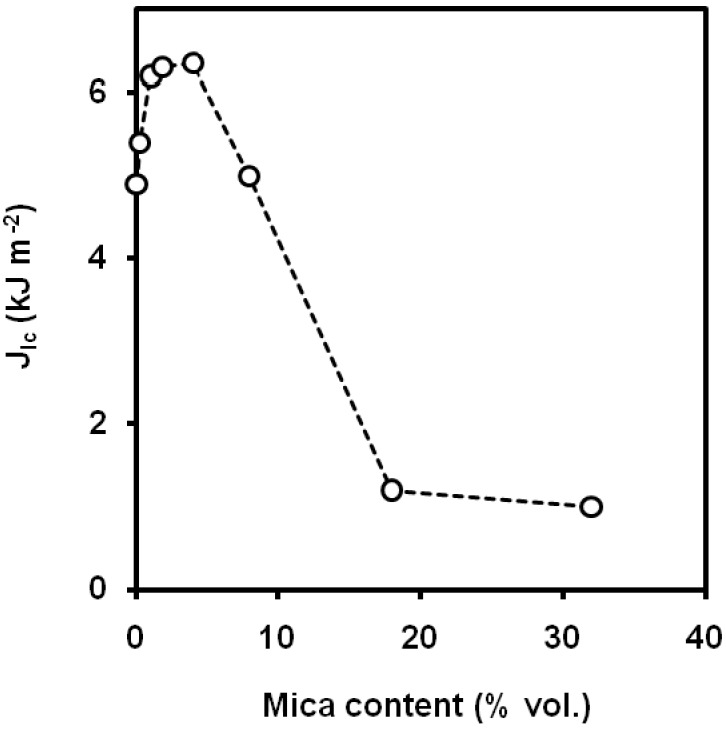
Influence of mica content on the values of *J_Ic_* (adapted from [111]).

#### 5.2.3. Mica

Vu-Khanh *et al*. [111] observed an enhancement of *J_Ic_*, however, only for filler volume fractions lower than 10 vol.% (Figure 32). Mica orientation had not a significant effect on the crack growth resistance of the composite, but mica flake size with smaller average size led to a decrease of the maximum crack growth resistance. Surface treatment with silane reduced the values of crack growth resistance. Khonakdar *et al*. [112] reported a decrease of the Izod impact strength with the filler content, whereas the addition of MAPP [113] and the surface treatment of mica with a silane coupling agent did not give significant differences. On the contrary, Chiang *et al*. [114] observed that the surface treatment of mica with silane coupling agent combined with acrylic acid, led to a thick interlayer that resulted in acceptable impact properties.

### 5.3. PP Composites with Short Fibres

Fundamental work on the toughness of short fibre composites (by ‘short’ we mean ‘non-continuous’, a definition that needs no specific reference to the concept of fibre critical length, l_c_) has been performed by many researchers, and developed extensively in the group of Lauke [115,116,117,118,119]. Avoiding the complexities of the energy calculations arising from all the fracture mode contributions [119], there is much evident that the fracture toughness of these particular kind of composites is often dominated by the fibre pull-out, although other processes such as fibre debonding are sometimes significant as well. The particularity of this kind of composites has pushed scientists to find theories that try to correlate microstructure and fracture toughness. Two concepts are the most widespread for the description of the toughness in terms of fracture mechanics: the microstructural efficiency concept of Friedrich [120] and the total fracture toughness concept of Lauke *et al*. [117] and Kim and Mai [121]. According to the microstructural concept, the relative fracture toughness is linearly related to the microstructural efficiency factor (*M*):

(14) $$\frac{K_{c,c}}{K_{c,m}}=a_{m}+n\;\Omega \;\equiv M$$

where *a_m_* is the matrix stress condition factor, *n* is the energy absorption ratio and *Ω* is the reinforcing effectiveness parameter. By other hand, the constitutive model on the work of fracture of composites by Lauke considers debonding, sliding and plastic deformation of the matrix in the dissipation zone and pull-out and matrix fracture in the process zone. The physical basis and the equations related to the individual modes of failure are reviewed by Kim and Mai.

**Figure 33:**
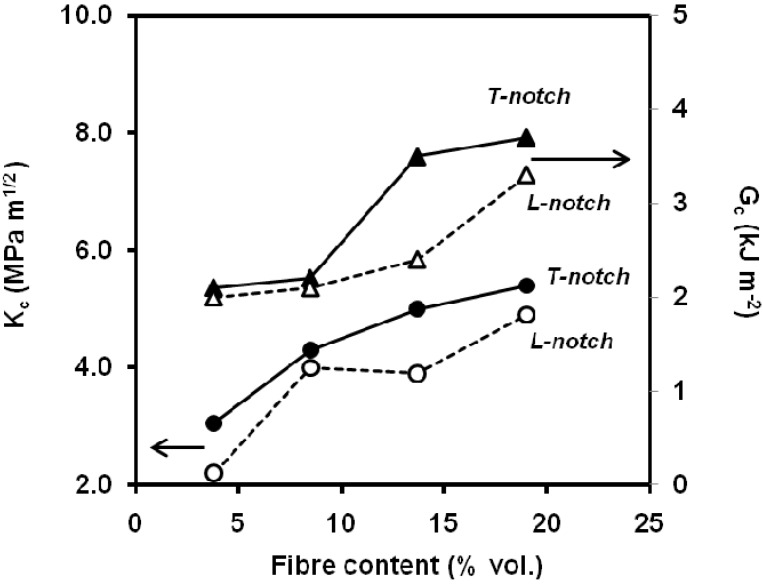
Influence of short glass fibre content on the values of *K_c_* and *G_c_*. *L* and *T* denotes notch longitudinal and transversal to the melt flow direction, respectively.(adapted from [124]).

**Figure 34:**
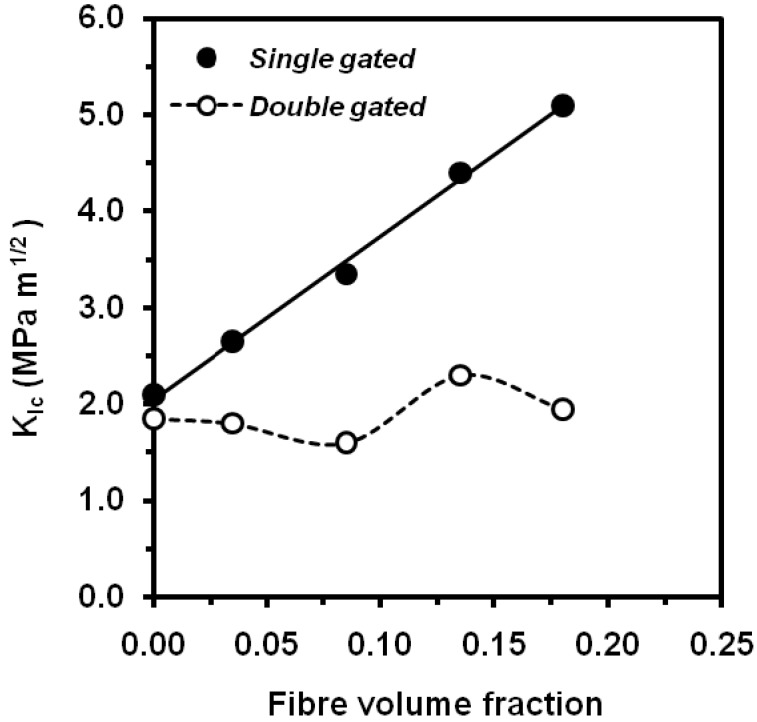
Influence of short glass fibre content on *K_Ic_* values for injected-moulded PP composites.(adapted from [129]).

It has been reported an increase of the values of critical stress intensity factor and the critical energy release rate with the short glass fibre content at low strain rate [122,123] and at impact speed [124,125] (Figure 33). This increasing trend (fibre content and fibre transverse orientation) has also been observed for cellulose fibre in instrumented Charpy impact testing [126,127] the obtained values of fracture toughness for PP/cellulose fibre composites showed a good correlation between microstructure and fracture toughness according to the Friedrich’s microstructural efficiency model [120]. Nevertheless, Fu *et al*. [128] have noticed that notched Charpy impact energy of short glass fibre and short carbon fibre-PP composites increases up to a maximum of ca. 8 vol.% and then stabilizes. For single short glass and carbon fibre composites, the mean fibre lengths decreased with an increase of the fibre volume fractions due to enhanced fibre-fibre interaction. This eventually resulted in the insensitivity of the notched Charpy impact energies of composites to the fibre volume fraction. As the mean fibre length depends on the processing condition, so does Charpy impact energy. Onishi *et al*. [129] report an increasing trend of the *K_Ic_* values obtained from SENT specimens obtained by single-gated mouldings (Figure 34), but the presence of weldlines in double-gated mouldings reduced fracture toughness by as much as 60% for composite containing 40% by weight short glass fibres.

**Figure 35:**
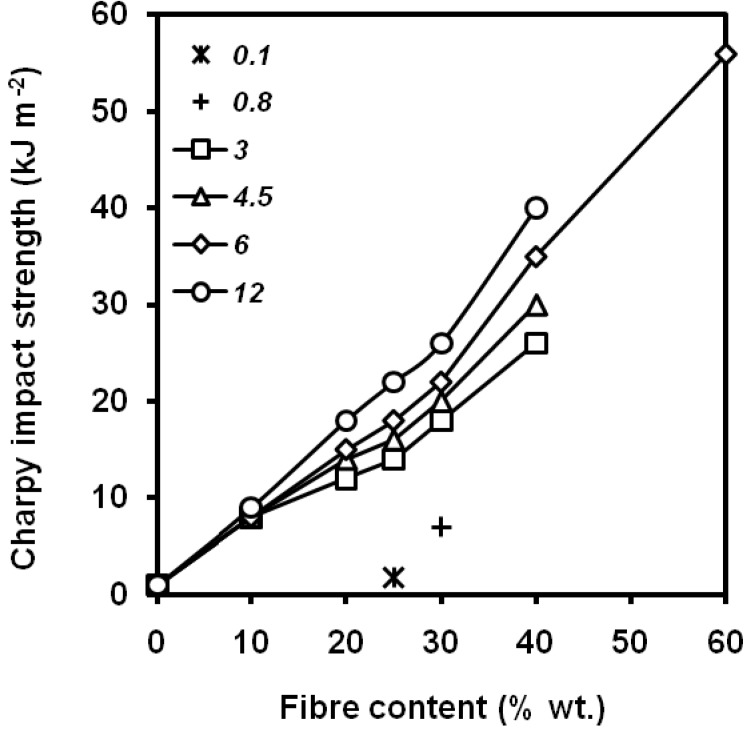
Influence of short glass fibre content and fibre diameter (0.1-12 mm) on notched Charpy impact strength of PP/short glass fibre composites (adapted from [125]).

The fibre length plays also a decisive role in the fracture performance of PP/short fibre composites. Thomason *et al*. [125] showed in PP/short glass fibre composites, that the longer fibre length the greater values of notched Charpy impact strength, within the range 0-12 mm of fibre lengths (Figure 35). A dependence of the fibre orientation must be also taken into account [130].

With respect to the fibre-matrix interfacial adhesion, Karger Kocsis *et al*. noticed that at static fracture the decrease in the *J*-integral *vs.* fibre volume fraction was smoother at poor glass fibre/PP bonding [131]. Analogously, the dynamic *J*-integral increased with the fibre content, but its critical value was enhanced with bonding, coupling glass fibre and PP by using MAPP. The above controversy can be explained by considering the facts that fibre surface treatment induces changes in the interface region. At dynamic fracture the fracture toughness increased with fibre content, more pronounced when the glass fibre was not coated with aluminium [132].

## 6. Fracture Toughness Characterization of PP Nanocomposites

For the last ten years or so there has been considerable interest in polymer nanocomposites and particularly in nanoclay composites. Functional properties such as fire and moisture permeability resistance, and some specific mechanical properties such as creep and wear resistance are the driving forces for the development of polymer nanocomposites. However, nanocomposites need sufficient stiffness, strength and toughness for their particular design purpose [133]. There is a tendency to believe that all the properties of composites must be enhanced if the particle size is very small. Small particle size has a positive effect on many of the functional properties of polymer composites, and as previously seen, toughness is particle size dependent. There are many toughening mechanisms in composites which cannot be effective with nanoparticles. For example nanoparticles are too small to cause significant crack bridging or crack deflection. On the other hand the very large surface area of nanoparticles does provide the possibility of large energy absorption if they delaminate. However, even here there is an optimum particle size for toughening because the stress necessary to cause delamination is inversely proportional to the square root of the particle size.

**Figure 36:**
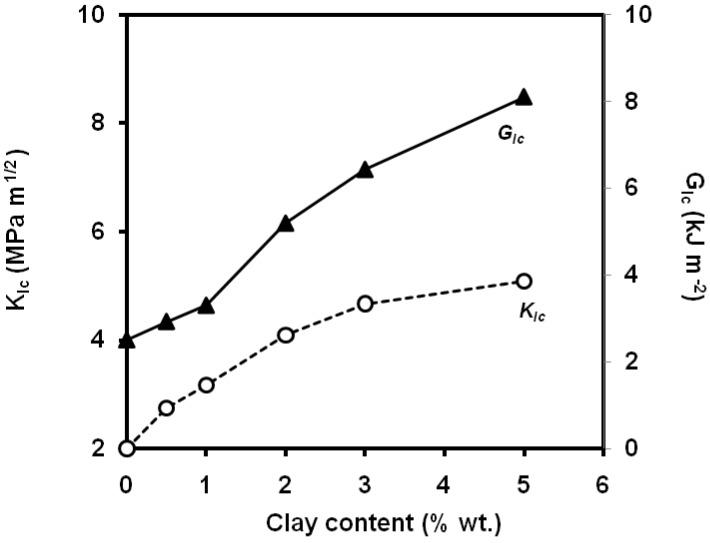
Influence of montmorillonite content on *K_Ic_* and *G_Ic_* values composites.(adapted from [134]).

**Figure 37:**
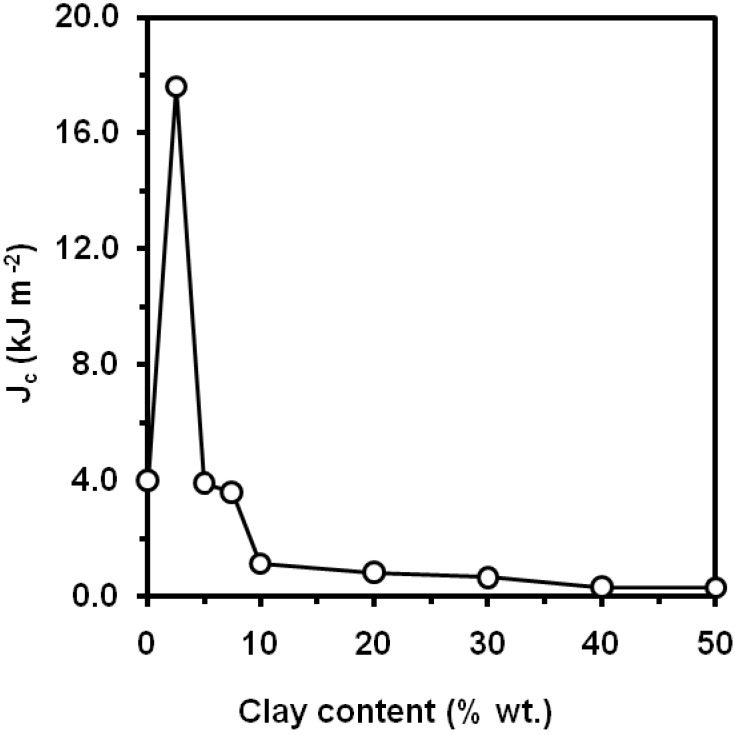
Influence of octadecylamine surface-treated montmorillonite content on *J_Ic_* values (adapted from [135]).

Kanny *et al*. [134] report that the stress intensity factor and strain energy release increased with the addition of commercial montnmorillonite (Figure 36), exhibiting a maximum improvement for a clay content (montmorillonite) of 5 wt.% This improvement is attributed to the presence of intercalated nanoclay structures in the PP nanocomposite structure that acted as load-bearing agents, and also acted as crack stopping agents. Chen *et al*. [135, 136] report a dramatic increase in *J_Ic_* from 4 kJ m^-2^ for the unfilled MAPP to about 17 kJ m^-2^ for 2.5 wt.% of montmorillonite surface-treated with octadecylamine (Figure 37), although they report a moderate increase in the tearing modulus, as indicated by Cotterell *et al*. [133] and these value should be taken with caution. Higher clay contents cause particle agglomerates and thus a reduction of the fracture toughness is observed. Kim *et al*. showed a decreasing trend of the Izod impact with the montmorillonite content [137]. A maximum at 2 wt.% of carbon nanotubes within the range 0-5 wt.% was found in the values of notched Charpy impact strength [138].An optimum of *J_c_* values at 5 wt.% of CaCO_3_ nanoparticles was reported by Kosh *et al*. [139], two times higher than those of neat PP. Wang *et al*. [140] reported an optimum value of notched Charpy impact strength at 10 wt.% CaCO_3_, and then the impact strength of the nanocomposites decreased with increasing filler loading, but it still reached at 20 wt.% of calcium carbonate, a value five times than that of neat PP.

**Figure 38:**
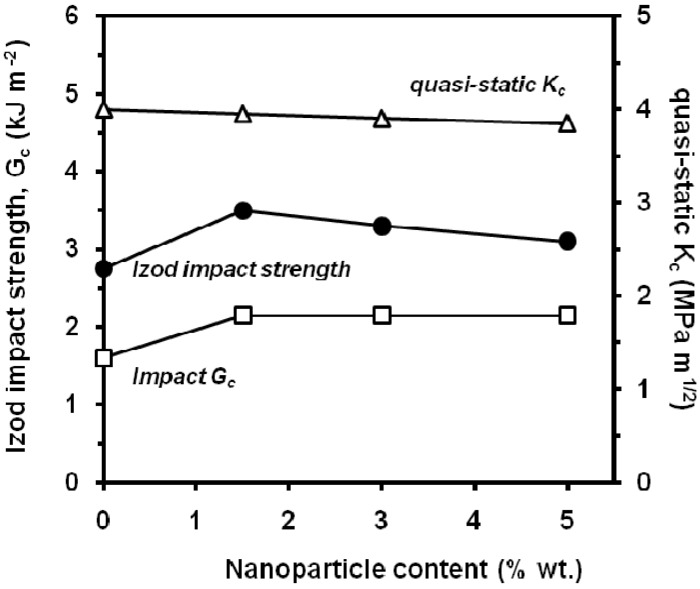
Effect of aluminium oxide nanoparticles on quasi-static and dynamic fracture parameters(adapted from [141]).

Zhao *et al*. [141] found in composites with aluminium oxide, Al_2_O_3_, nanoparticles (Figure 38) that at quasi-static loading rate, the fracture toughness was found nearly unvaried with the filler content. Under impact loading rate, the notched Izod impact strength and the impact fracture toughness indicate that the impact fracture toughness increases initially with the addition of 1.5 wt.% of Al_2_O_3_ nanoparticles into the PP matrix. However, with the further addition of up to 3.0 and 5.0 wt.% Al_2_O_3_ nanoparticles, both notched Izod impact strength and impact *G_c_* decreased slightly. Crazing and microcracking with dilatational feature were found to be the main fracture mechanisms for the virgin PP and the Al_2_O_3_/PP nanocomposites.

**Figure 39:**
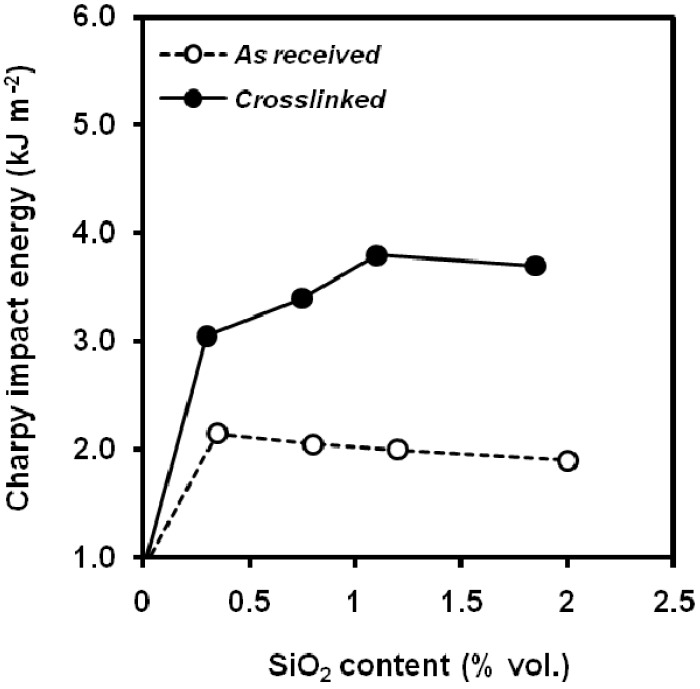
Effect of SiO_2_ crosslinking on notched aluminium oxide nanoparticles on quasi-static and dynamic fracture parameters(adapted from [144]).

The surface treatment of montmorillonite with sodium salt of alkylammonium within the range 1-3 wt.% [142] resulted in values of notched Izod impact strength of 11-12 kJ m^-2^, higher than that showed by unfilled PP, ca. 4 kJ m^-2^. Polypropylene containing 4 wt.% montmorillonite clay surface-modified with dimethyldialkylammonium improved the Izod impact behaviour in the temperature range of 0 to 70 ºC, observing differences in the fracture surfaces [143]. The fracture initiation and propagation of neat PP was characterized by crazing and vein-type features, whereas the reinforcement of PP with nanoclay alters the primary mechanism of plastic deformation from crazing and vein-type to microvoid-coalescence process. Silane-treated silica nanoparticles were blended by using in-situ cross-linking method [144] leading to a enhanced filler/matrix interaction and thus the volume fraction of interphase was increased, conducting to an improvement of the Charpy impact strength (Figure 39). Zhang *et al*. [145] studied the fracture toughness of nanocomposites of CaCO_3_ through the reflective optical caustics method, a way to evaluate the stress singularities at the crack tip because of its simple optical patterns, which can establish the relation between the stress field parameters and the maximum transverse diameter of caustic curve. They found nanocomposites of CaCO_3_ with a non-ionic modifier, polyoxyethylene, increased the values of the stress intensity factor. The absence of the non-ionic modifier gave smaller values**.** The addition of POE-g-MA in PP/CaCO_3_ nanocomposites [140] increased the impact strength but reports that the addition of PP-g-MA or EVA-g-MA was detrimental to the impact strength. A positive effect of the surface treatment of calcium carbonate nanoparticles with stearic acid on the notched Izod impact strength is reported [146].

Bureau *et al*. modified the interfacial adhesion PP-clay through the addition of surface-treated montmorillonite and maleic anhydride-grafted polypropylene [147] and studied the fracture behaviour by the EWF methodology (Table 8). Clay particles were found to act as void nucleation sites within the PP matrix, which led to higher void nucleation, reduced void growth and rapid void coalescence, accompanied by extensive fibrillation, causing an important reduction in fracture toughness with respect to PP, but also an important increase in plastic work dissipation. The presence of grafted PP increased notably the values fracture toughness. It has been also reported that the molecular weight of MAPP had an effect on the quality of clay particle dispersion [148], the better the lower molecular weight; a better dispersion provided higher values of specific work of fracture, *w_e_*. Saminathan *et al*. [149] studied also a system PP/MAPP/montmorillonite through the EWF, with clay content 5 wt.% and found an increase in the values of *w_e_* from 23.3 kJ m^-2^ for pure PP to 29.3 kJ m^-2^ for the nanocomposite.

**Table 8:** EWF parameters of PP nanocomposites with differences in the interfacial adhesion between phases (adapted from [147]).

Matrix	Clay	MAPP	w_e_ (kJ m^-2^)	βw_p_ (MJ m^-3^)
PP			14.9	0.38
PP	2% Cloisite 15A		3.8	1.29
PP	2% Cloisite 15A	4% Epolene E43	7.0	1.63
PP	2% Cloisite 15A	4% Polybond 3150	6.8	1.59
PP	2% Cloisite 15A	4% Polybond 3150	15.9	0.65
PP	2% Cloisite 30B	4% Polybond 3150	12.9	1.19

## 7. Conclusions

Filled-polypropylene composites are material able to fulfil a satisfactory balance of mechanical properties for several aims in the engineering field (e.g. automobile, furniture, household, etc.). An accurate selection of the type and content of filler, as well as its processing, allow polypropylene to become a competitive material with respect to engineering plastics in terms of fracture strength.

Besides the normalised tests which simply measure the impact strength, several theories have been developed to explain the fracture behaviour and determine fracture toughness values of plastic materials and composites. Thus, in PP composites the LEFM is applied at high strain rates, whereas other theories as *J*-integral and EWF deal with the cases in which the material develops a certain extent of plastic deformation, as usual in fracture at low strain rate. The Fracture Mechanics aims to explain the fracture behaviour in the beginning and define its characteristic parameters. Ideally, the fracture parameters should be independent of the testing geometry and specimen dimensions. However, fracture toughness is one of the mechanical characteristics that have more difficulty in its determination and analysis, as there are numerous factors involved: temperature, strain rate, specimen dimensions and testing geometry, mainly. This joins to the influence that on the mechanical properties of PP composites has the polymer type (*i.e.* homopolymer or copolymers), content, size, shape, nature and surface treatment of the particles employed as reinforcement.. This complexity comes to being in published Works about similar systems. In this paper, the state of art of the characterization of fracture toughness in particulate filled PP composites has been reviewed, from a point of view theoretical, methodological and experimental. It’s still necessary to deep in the establishment of a clearly defined criterion for the quantification of toughness in this kind of composites.
